# Implementing conjunctive management of water resources for irrigation development: A framework applied to the Southern Plain of Western Nepal

**DOI:** 10.1016/j.agwat.2023.108287

**Published:** 2023-06-01

**Authors:** Vishnu Prasad Pandey, Nirman Shrestha, Anton Urfels, Anupama Ray, Manohara Khadka, Paul Pavelic, Andrew J. McDonald, Timothy J. Krupnik

**Affiliations:** a Department of Civil Engineering, Pulchowk Campus, Institute of Engineering, Tribhuvan University, Lalitpur 44600, Nepal; b Center for Water Resources Studies (CWRS), Institute of Engineering, Tribhuvan University, Lalitpur 44600, Nepal; c International Water Management Institute (IWMI) – Nepal, Lalitpur 3, Pulchowk, Kathmandu, Nepal; d International Maize and Wheat Improvement Centre (CIMMYT) – Bangladesh, Dhaka 1213, Bangladesh; e CIMMYT, South Asia Regional Office, Lalitpur, Nepal; f Wageningen University and Research, Wageningen, Netherlands; g Centre of Research for Environment Energy and Water (CREEW), Baluwatar, Kathamandu - 4, Nepal; h IWMI – Vientiane, Lao PDR, Laos; i Cornell University, 617 Bradfield Hall, Ithaca, NY 14853, United States

**Keywords:** Climate change, Conjunctive use, Conjunctive management, Groundwater, Water policy

## Abstract

Climate variability and insufficient irrigation are primary constraints to stable and higher agricultural productivity and food security in Nepal. Agriculture is the largest global freshwater user, and integration of surface- and ground-water use is frequently presented as an strategy for increasing efficiency as well as climate change adaptation. However, conjunctive management (CM) planning often ignores demand-side requirements and a broader set of sustainable development considerations, including ecosystem health and economics of different development strategies. While there is generic understanding of conjunctive use, detailed technical knowhow to realize the CM is lacking in Nepal. This article presents a holistic framework through literature reviews, stakeholders consultations and expert interviews for assessing CM and implementation prospects from a systems-level perspective. We demonstrate the framework through a case study in Western Nepal, where climatic variability and a lack of irrigation are key impediments to increased agricultural productivity and sustainable development. Results show that knowledge of water resources availability is good and that of water demand low in the Western Terai. Additional and coordinated investments are required to improve knowledge gaps as well as access to irrigation. There is therefore a need to assess water resources availability, water access, use and productivity, to fill the knowledge gaps in order to pave pathways for CM. This paper also discusses some strategies to translate prospects of conjunctive management into implementation.

## Highlights

• Customized framework development for assessing prospects for conjunctive management.
• Conjunctive management status in western Nepal assessed by applying the framework.
• Knowledge on water availability and demand are moderate and low, respectively.
• Additional and coordinated investments required to improve access to irrigation.

## 1 Introduction

Global climate change adaptation in low-income nations hinges on finding profitable and resilient water management methods for agriculture (Miralles-Wilhelm, 2021). Agricultural transformation can help move low-income nations to middle-income by 2030, as the agricultural sector in such countries accounts for more than 25% of gross domestic product (GDP) (World Bank, 2021). Nepal's net farmed land is 48% irrigated, while only 39% has year-round irrigation (IMP, 2019). Iirrigated agriculture can contribute in achieving the Sustainable Development Goals (SDGs) (Medici and De Wrachien, 2016) such as SDG-1, SDG-2 and SDG-13, as irrigation can be associated with inclusive growth, food security, and climate resilience. For example, irrigating rain-fed systems can enhance wheat (34 ± 9%) and maize (22 ± 13%) yields worldwide (Wang et al., 2021), buffering production against dry spells and drought thus affecting food availability and costs (Namara et al., 2010). Irrigation's year-round benefits dwarf the prior statistics (Namara et al., 2010). However, increasing physical and economic water shortages challenge to sustainable and equitable access to water in agriculture (Tran and Weger, 2018, among others), with arid areas facing severe difficulties.

In response to these pressures, the conjunctive use (CU) and conjunctive management (CM) of surface- and ground-water resources is a frequently cited principle for adapting agricultural water management to climatic change (Foster et al., 2010, Evans et al., 2014, Van der Gun, 2020). CM refers to coordinated surface- and ground-water management and CU refers to the efficient use of both resources (van Steenbergen, 2015). Following the definition of van Steenbergen (2015), this paper uses the term ‘CM’ to discuss the co-management of both the resources within its context. Although ample evidence exists of integrated impact assessments on the one hand (Bejranonda et al., 2013, Afshar et al., 2021) and some governance guidelines on the other hand (Hamamouche, 2017; Foster et al., 2010), the appropriate framework for assessing and implementing CM remains largely absent. This paper presents an integrated framework for assessing and implementing CM in agriculture to address this knowledge gap, through a case study approach to irrigation development in Western Nepal. The region has significant irrigation development projects underway for surface- and ground-water, highlighting its importance for achieving SDG6 and SDG13. All four priority irrigation projects in Nepal designated as National Pride projects are located in Western Nepal (NPC, 2021). Significant ongoing private investments in groundwater irrigation highlight the need for better access to water among the region’s farmers (Urfels, 2020).

In the context of a lack of a comprehensive methodology for evaluating CM practice, this study aims to answer following questions: (1) How has CM planning literature evolved over time and what might be an appropriate conceptual framework for assessing status of CM in an area? (2) What is the status of CM in Western Nepal as reflected by appling the newly developed framework? (3) What considerations or strategies are required to implement the CM framework in practice?

## 2 Development of a methodological framework for CM planning and implementation

### 2.1 Evolution of CM as an irrigation policy tool

Consumptive use has been a water management approach to achieving socio-economic benefits since the 1960s (Bhattarai and Shakya, 2019). Vincent and Dempsey (1993) described how, since then, CM has developed as a tool for water resources planning and irrigation scheme management, joining the World Bank's irrigation policy in the early 1980s after Pakistan's agricultural productivity increased by 20%. China (Liu, Hu, and Chen, 2020), Algeria (Hamamouche, Kuper, Riaux, and Leduc, 2017), Australia (Ticehurst and Curtis, 2019), Spain (Escalante and Sauto, 2018), and India (Dillon et al., 2009) all use CM for irrigation. However, different sectors/stakeholders interpret CM differently. This paper defines CM as a planned and coordinated integration of surface- and ground-water use/management over time and space in order to facilitate irrigation, applicable to all scales of management, to optimize productivity and achieve environmental sustainability and equity (Bhattarai and Shakya, 2019, Evans et al., 2014, FAO, 1995, Foster et al., 2010, Hamamouche et al., 2017, Vincent and Dempsey, 1993).

The basic principle of CM is to regulate surface- and ground-water use based on needs and objectives, and to optimize the distribution of both surface- and ground-water in a command area at a time (Liu et al., 2020). Hedging reduces environmental externalities (e.g., energy for deep pumping, non-attainment of ecosystem flow requirements with surface water diversions) and hazards such as crop failure or yield loss.

Instead of developing one source to make up for the mismanagement of another, CM seeks to maximize water use(s). By pumping groundwater during the dry season, it creates additional subterranean room to store surface water arising from infiltration during high flows for use in the next season. CM has many benefits that may improve socioeconomic results (Foster et al., 2010), but it depends on hydrological and hydrogeological conditions. It can buffer high flow in surface water supply systems in order to adjust to climate change and provide irrigation (Foster et al., 2010, Rao et al., 2004, Ticehurst and Curtis, 2019). It also lowers flood hazards, improves health and sanitation, and boosts environmental flows.

However, the CM approach demands the co-existence and economic availability of both surface- and ground-water resources. A surface water distribution system requires significant capital and operating costs, with its management demanding strong social organization. The CM-related interventions have varied advantages determined by governance, infrastructure, and water management (Evans et al., 2014). These can be engineered (aquifer storage and/or recovery), involve integrated water planning, and be bottom-up (farmer-led at the farm level), or top-down (a more strategic approach where inputs of both resources are managed/planned centrally by the government) (Holley et al., 2016).

### 2.2 Conjunctive management in practice

Nepal and most of South Asia have yet to fully implement CM planning. Meeting national and global goals such as the SDGs is hindered by the lack of a strategic vision for CM planning to support socio-economic outcomes (Foster et al., 2010). Optimizing surface- and ground-water use/management depending on the situation, requires major financial investment, infrastructure, and institutional conversion.

The CM's hydrological cycle connectivity is appealing (Holley et al., 2016). However, ‘joint use’ in irrigation projects mobilizes groundwater for many purposes rather than methodically optimising water use and conservation (Vincent and Dempsey, 1993). Though literature exists on the effects of ‘joint use’ on farmers and water management by significantly modifying irrigation system reliability while minimizing externalities, the technique comes at a cost – expenses which are reasonable if dependability and externalities improve. These factors rely on context. Both resources have CM concerns and information gaps, including:

• Irrigation command areas need to move from spontaneous CU to CM. However, an upper limit on groundwater abstraction should be recognized for CM in irrigation, which varies with the scenario of surface water delivery and hydrogeological setting of an area (Foster and van Steenbergen, 2011). Although farmers use groundwater to supplement surface water deficit by default, where the possibility exists, the CM needs to be considered at the system scale rather than only at the farm scale. It requires a realistic estimate of water demand and availability.
• The constraints for planned and coordinated CM are related to various aspects such as water resource availability and physical characteristics of water resources (Cleaver, 2012), physical infrastructure, and operational institutions (Foster and van Steenbergen, 2011), in addition to environmental and social/equity issues (Hamamouche et al., 2017).
• Failure to characterize existing and potential irrigation institutions, critical assessment of their functioning, and exploring their linkages and information exchange mechanisms is not only a fundamental limitation for planned and coordinated management of surface- and ground-water resources but also for exploring prospects for improved policies. Addressing this challenge requires efforts to coordinate across actors and sectors (e.g., individuals, distinct water sources potentially governed by different authorities, etc.) (Blomquist, Schlager, and Heikkila, 2004).
• There are equity issues in CM, with difficulty in securing equal/equitable access rights to and ownership for to all sections of society over surce- and ground-water resources (Khadka et al., 2021).
• Not all areas have equal prospects of achieving CM. Characterizing current CM practices, synthesizing learnings, and assessing the prospects for planned CM provides an adequate basis for workable plans to achieve it.
• Achieving effective CM requires various strategies such as planned investments in hardware (e.g., modernization), software (digitized and real-time resources monitoring and improved databases), and planning and management capacities. It may entail institutional reform (Shah et al., 2006). Optimizing CM also requires providing equal attention to the capacity building of irrigation system managers and farmers.

### 2.3 A framework for CM planning and implementation

**Fig. 1:**
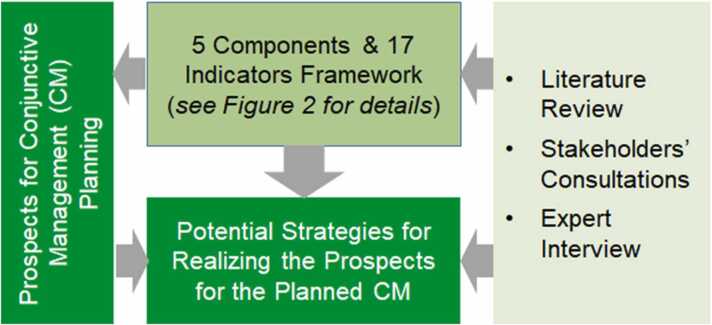
Methodological framework for assessing prospects and implementation for planned CM.

The overall framework (that is, a basic structure underlying the concept) for assessing the prospects for CM planning and implementation was developed based on literature review, stakeholder consultation and expert interviews. It consists of components of assessing prospects as well as identifying potential strategies for realizing the prospects for the planned CM (Fig. 1). Each of the components is elaborated in the following sub-chapters.

#### 2.3.1 Assessing prospects for CM planning

**Fig. 2:**
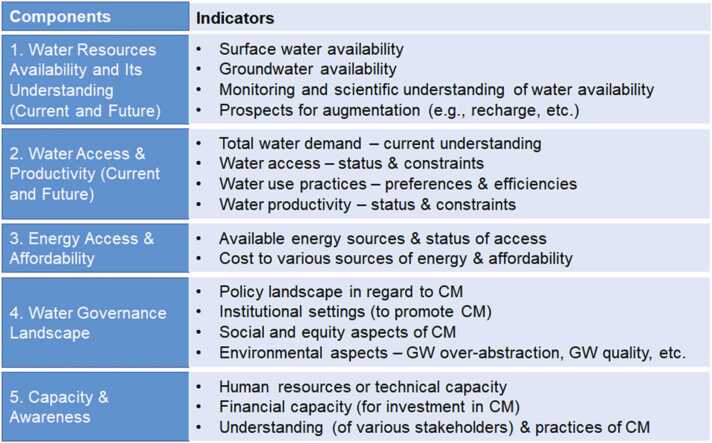
A five-component and 17-indicator methodological framework for assessing prospects for CM.

Based on insights from the literature and engagement with stakeholders through virtual workshops, a five-component and 17-indicator methodological framework (Fig. 2) has been developed to assess prospects of the CM in the study area and address knowledge gaps identified in the earlier section.

Most of the accessible literature across the globe was reviewed rigorously to characterize various aspects of the CM to be considered in identifying the prospects. The relevant literature was collected, screened, and examined to synthesize such initiatives. A Google search using selected key words (e.g. hydropower project, irrigation project, water supply project, Western Nepal, Karnali basin, water development programme) found existing and proposed water development projects in USAID’s Feed-the-Future Zone of Influence (FtF-ZoI) area in Sudurpashchim and Lumbini Provinces. The websites of the following government departments provided project information: Department of Energy Development (DoED), Bheri Babai Diversion Multipurpose Project, Water Resources Project Preparatory Facility, Water and Energy Commission Secretariat (WECS), Nepal Electricity Authority, and Department of Water Supply and Sanitation Management (DWSSM). For project updates, project-related websites (e.g., Digo Jal Bikas, The Rising Nepal, Prime Minister Agriculture Modernization Project, My Republica, etc.) in Nepali and English language were searched. IMP, 2018, IMP, 2019 were also reviewed. This identified 35 articles/papers.

Reviewing abstracts and paper contents was the first step to screen the literature. Then, literature on existing and proposed projects, news updates, and evidence of a report providing crucial information on the water project were chosen. Screening the literature yielded 28 studies. Eight were then removed because they provided general information for a small area or a thorough engineering design. Some material was perception-based or review-based, therefore original and latest reports were followed. Finally, 19 items of literature were chosen for extensive review and synthesis.

A preliminary framework was shared with stakeholders during a virtual workshop with more than 25 participants, including a mix of representatives of government agencies (policymakers and implementing departments), non-government agencies, the private sector, research/academic sector, and local government and community representatives. Well ahead of the workshop, the draft methodology was shared with the participants, who were briefed over the phone about the framework and their consent obtained for paraticipation in the workshop. On the day of the workshop, the methodology was presented briefly, after which each stakeholder in turn was requested to share their thoughts turn-by-turn on suitability of indicators, availability of data, and ways of implementing the framework. The methodology/framework was finalized after incorporating the stakeholder inputs.

Although the climate crisis is not explicitly spelled-out in the indicators and components, they are already embedded in the components and indicators related to water resource availability and water demand, as climate change can affect both of these (e.g., Pandey et al., 2019, Pandey et al., 2020b). Future water demand is associated with both climatic and non-climatic factors (such as land use/cover change, economic growth, potential changes in agricultural and food security policies).

Both primary and secondary data were collected, pre-processed and analysed. Results were validated in consultation with stakeholders, following the approach suggested in Ticehurst and Curtis (2019). Secondary data include hydrological and meteorological data collected from the hydro-met department of Nepal and published literature. Primary data includes situation assessment on various aspects of CM (e.g., status of monitoring, prospects for augmentation, water use practices, human resources and technical capacity) through engagement with a well-mixed group of stakeholders.

#### 2.3.2 Identifying workable strategies to realize the prospects

The Western Plains (the Terai) of Nepal was selected as a case study area for operationalising the framework, and after a rigoous literature review the current status of all the indicators was synthesized and potential strategies identified for realizing the prospects for the CM. These were further streamlined and finalized in consultation with various stakeholders and taking into consideration suitability for the study area.

## 3 Context and description of the case study area: Western Nepal’s Southern Plain

The Southern Plain in Western Nepal, part of the South Asia’s Indo-Gangetic plain, is among Nepal's most impoverished and food insecure regions and is thus crucial for progress towards SDG1 and SDG2. At the same time, this region has already experienced climate change impacts on water availability (Pandey et al., 2019, Pandey et al., 2020b) and is prone to drought.

### 3.1 Agriculture and water management in Nepal

Agriculture production is a decisive factor in the sustainable development of Nepal’s socio-economy.It engages more than 60% of the population and is the second-largest contributor to GDP (27%); however, the sector’s productivity is poor (IFAD, 2020). Nepal has a relatively abundant amount of water resources (99.3 Mm^3^/ha of irrigable land, 7707 m^3^/capita/year) (data source: irrigable land area from Irrigation Master Plan; water resources availability from MoEWRI (2020); population from CBS (2021)), however, according to government statistics only 48% of the net cultivated area has some-sort of irrigation facility and only 39% has year-round-irrigation (YRI) (IMP, 2019). Furthermore, economic development and population growth increase the water demand of agriculture and other sectors, in turn increasing the demand for water overall. At the same time, climate change is expected to lead to a more erratic water supply (WECS, 2011). Enhancing access to irrigation has therefore become a key challenge for the sustainable development of agriculture in Nepal and this can be addressed through the integrated use of surface- and ground-water resources, or through CM. Although CU is happening by default in southern Nepal, because it is a surface water-rich country, CM is typically left out of the picture or marginalized in policy and plans (Upadhyay and Gaudel, 2018). In this context, CM can be considered as an important pathway for enhancing access to irrigation and prioritizing investments because of its prospects to enhance water-use efficiency, improve water security, and ultimately result in better water productivity, along with several other benefits (e.g., flood management) (Pandey et al., 2021). Furthermore, CM can effectively maximize water use benefits through augmenting supply, increasing productivity, avoiding aquifer mining, and integrating surface- and ground-water management (Mattiuzi, Marques, and Medellín-Azuara, 2019).

The Irrigation Master Plan of Nepal (IMP, 2019) mentions that out of 48% of irrigated land in Nepal, 19% is irrigated by CU (not CM!), which is primarily spontaneous (unplanned, unregulated and unmanaged) (Foster and van Steenbergen, 2011; Pandey et al., 2021). However, CM, which focuses on government-led initiatives on groundwater development and use for a larger area, ideally with community participation, is still not receiving enough attention in Nepal in general and Western Nepal in particular. Nevertheless, the IMP (2019) established a bold goal to increase YRI from the current 39–55% by 2025, 66% by 2030, and 100% by 2045. Similarly, nearly 60% of cultivated land in the southern plain of Nepal, also known as the food basket of the country, has access to irrigation, most of it from groundwater (Urfels, McDonald, Krupnik, and van Oel, 2020). Still, groundwater resources in Terai’s alluvial aquifers are underutilized for various reasons, including energy constraints (access or affordability) that limit the farmers’ ability for groundwater pumping (Nepal, Neupane, Belbase, Pandey, and Mukherji, 2021). Under these circumstances, in the southern plain of the country, targets for YRI can be achieved only through CM.

### 3.2 Description of the study area

**Fig. 3:**
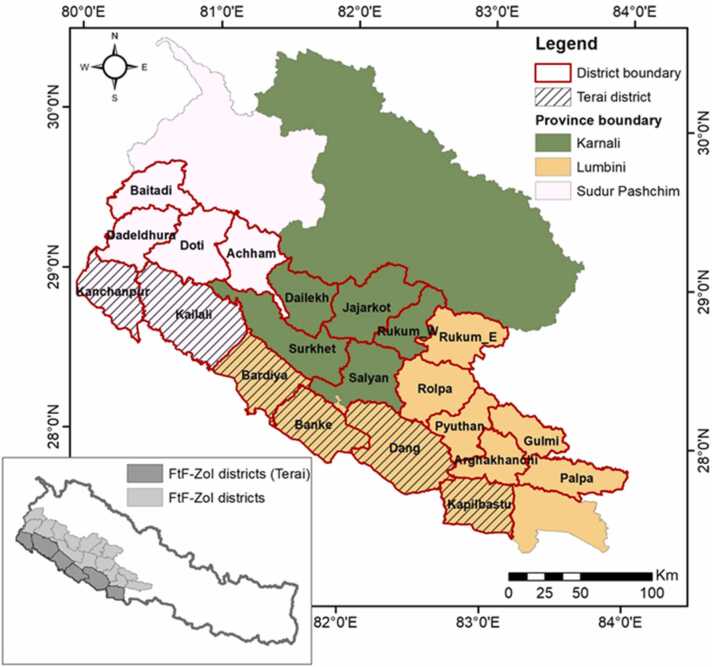
Study area in Western Nepal.

This case study focuses primarily on the FtF-ZoI, which spans 237 Palikas in 21 districts in three provinces in western Nepal. Six of those districts are in the Terai (Fig. 3), which accounts for an area of approximately 1.22 Million hectares (Mha). FTF-ZoI is considered as one of the important areas in the nation with the ability to contribute to doubling growth through productivity improvement. About 6.9 million people live in this region, and roughly 21% are considered poor. (FTF, 2016).

Both surface- and ground-water resources exist in Terai. Five river systems (i.e., West Rapti, Mahakali, Karnali, Mohana, and Babai) in the region provide surface water resources. While other rivers from the Chure or Mahabharat mountains are rain or spring-fed, the Mahakali and Karnali are snow-fed perennial rivers. Climatic factors, such as drought and heat stress, impact the FtF-ZoI in the Terai, which lowers the productivity of the local farming systems. Utilizing available water resources sustainably through CM may be a feasible approach to the problem; however, this will be determined by the economics of canal development, operation, and maintenance. The FtF-ZoI has a diverse socio-economy and culture. More than half of the population in the study area are smallholder farmers (< 0.5 ha landholding) or are landless, with the major source of income being a seasonal migration and rain-fed agriculture (Khadka et al., 2021).

## 4 Prospects for planned CM in Southern Plain of Western Nepal

In various irrigation systems in the Terai region of Nepal, spontaneous/unplanned CU is already in practice. Water managers and other essential stakeholders have not had enough policy conversations on CM, however, the FtF-ZoI areas offer a wealth of options and prospects for CM because of their high recharge capacity, accessibility to surface water supplies, and potential for high agricultural productivity due to their fertile fields. This section is divided into five sub-sections, each defined by a set of indicators as per the framework for analysing prospects (Fig. 3). The overall status of each indicator is summarized in tabular form in SM-1.

### 4.1 Water resources availability and understanding

#### 4.1.1 Surface water availability

**Fig. 4:**
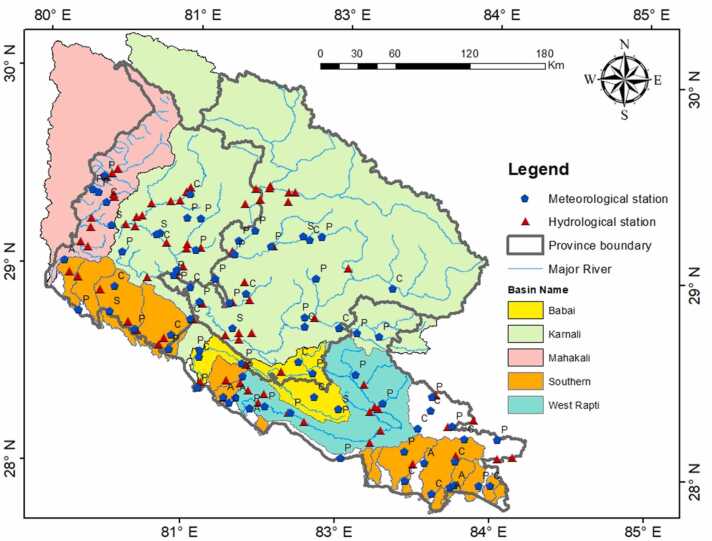
River systems and hydro-meteorological stations in the study area.

There are five river systems in the FtF-ZoI region (Fig. 4). The largest is the Karnali, which has several sub-basins (that is, West Seti, Bheri, Tila, Humla Karnali, and Mugu Karnali) and drains around 50,000 km^2^ of land up to the Indian border. Many southern rivers are seasonal, but during the rainy season, they constitute a significant water source for cultivation. These flow levels of those rivers can decrease dramatically during the non-monsoon season, as they mostly depends on monsoon precipitation (Shrestha et al., 2018). Due to their seasonal nature and low flow during the dry season, the rivers are not ideal for an irrigation supply without surface storage. According to observations, the Terai's numerous tiny southern rivers provide 13% of the nation's average flow, compared to 9% from the medium basins and 78% from the large ones (WECS, 2011). Studies specifically on southern rivers of Western Nepal are not accessible.

Key hydrological features and surface water availability (both current and future) of the major basins/sub-basins are reported in several literature (e.g., WECS, 2011; Dhami et al., 2018; Tripathee, 2018; Mishra et al., 2018; Bhatta et al., 2020; Dahal et al., 2020; Pandey et al., 2010, Pandey et al., 2019, Pandey et al., 2020a, Pandey et al., 2020b; etc.) and provided in SM-2a. Specific discharge of the basins/sub-basins vary from 18.8 l/s/m^2^ in Upper Karnali (above the Lalighat hydrological station) to 52.1 l/s/m^2^ in Mohana (SM-2b). As tabulated in SM-2a, average annual water availability in the Karnali River Basin, above Chisapani hydrological station (A = approx. 42,500 km^2^), is projected to increase by 8.4% and 10.9% under RCP4.5 and 8.5 scenarios, respectively, in far-future (2070–2099).

The current level of understanding of surface water availability is relatively good. Although the total amount of surface water passing through the FtF-ZoI is abundant, strong seasonality, spatial distribution, and topographical differences between river reach and agricultural areas have posed challenges for various beneficial uses. Using groundwater available in the Indo-Gangetic alluvial aquifer system in conjunction with surface water could be an appropriate strategy for the study area.

#### 4.1.2 Groundwater availability

Within the FtF-ZoI, all the Terai districts, except Dang have flat topography. Due to its abundance and high quality, groundwater is the Terai region's most significant water source. Nepal’s Terai and Inner Terai regions are thought to have abundant groundwater resources, although they are underdeveloped (Pathak, 2018). It is predicted that 88% of the groundwater that might be extracted sustainably is not being used, leaving plenty of room to increase groundwater use for productivity growth (Urfels et al., 2020). Understanding aquifer systems, their hydrogeologic properties, and the availability and distribution (both spatial and temporal) of groundwater is essential for the sustainable exploitation and management of groundwater resources. To characterize groundwater availability in the FtF-ZoI in Western Terai, this study discovered, screened, and ultimately reviewed 11 items of literature as detailed in Pandey et al. (2021).

There are primarily two types of aquifer systems in Nepal's Terai: shallow aquifers (0–46 m depth) and deep aquifers (> 46 m depth) (GDC, 1994). The aquifer characteristics and groundwater quality are provided in SM-3. The maximum aquifer yield from those aquifers vary from 167 to 2592 m^3^/day (see SM-3). Recharge from the Terai's northern boundary, the Bhabar Zone, along the Siwalik foothills, results in the Terai's renewable groundwater supplies (Shrestha et al., 2018), with rainfall and lateral inflows from the Bhabar zone directly infiltrating the Terai's aquifers, recharging them. Subsurface inflow and seepage losses through streams and rivers also provide significant inputs to the groundwater recharge. The Terai region of Nepal still lacks a clear delineation of groundwater recharge regions.

Analysis of data presented in IMP (2019), estimated based on projections of net groundwater recharge from a SWAT model, indicates that renewable groundwater availability (m/yr) across the FtF-ZoI districts varies from 0.11 (Dang) to 0.26 (Kailali), 0.33 (Banke), 0.38 (Bardiya), 0.42 (Kapilbastu) and 0.48 (Kanchanpur). The calculations also consider the relative contributions from the Terai, Bhabar, and seepage zones. According to WECS (2011), Dang's shallow aquifer alone has an estimated 130–140 MCM/yr groundwater availability. While groundwater abstraction for domestic use alone is estimated to be 7.43 MCM/year. Sapkota (2003) calculated the safe yield from the groundwater aquifers in the southern portion of Dang valley to be 31.6 MCM/year, indicating stress on the groundwater system. The actual volume of groundwater abstraction would be significantly larger after incorporating abstraction for irrigation. This shows that adequate planning and management are required to sustainably manage and use groundwater resources in the FtF-ZoI.

Few studies describe groundwater quality in the FtF ZoI, but there are issues with it, as indicated in SM-2. For example, of six Terai districts in Nepal, including Kailali and Kanchanpur in the FtF-ZoI, Yadav et al. (2012) analyzed the susceptibility of arsenic pollution. Out of the six study districts, the Kailali district has the highest mean arsenic concentration (6270 ppb), followed by Kanchanpur (4980 ppb).

#### 4.1.3 Monitoring and scientific understanding of water availability

Eighty-two hydrological stations, 41 precipitation stations, and 23 climatic stations monitor hydro-climatic conditions within and around the watershed (i.e., in three provinces in the FtF-ZoI area). Within the Terai districts of FtF-ZoI alone, there are 13 precipitation stations and seven climatic stations. Daily average values of hydro-met parameters are available at those stations, and data length and quality vary across the stations. The WMO guidelines suggest having one station every 600–900 km^2^ in the plain area as an ideal condition and every 900–3000 km^2^ as an acceptable condition (cited in Subramanya, 2013). An observation of just the Terai area of the FTF-ZoI indicates an adequate number of stations for monitoring precipitation in all Terai FtF districts except Kailali (i.e. rainguage density of Kapilbastu: 1656 km^2^; Dang: 1507 km^2^; Banke: 473 km^2^; Bardiya: 504 km^2^; Kailali: 3312 km^2^; Kanchanpur: 1633 km^2^).

Due to insufficient thorough hydrological modelling, scientific understanding of surface water resources is lacking for various basins, including West Rapti and Babai, including all water balance components and their spatio-temporal distribution over historical periods. In addition, these watersheds lack information on predicted future climatic changes and their effects on water supply.

Despite the abundance of information, the spatio-temporal distribution of groundwater resources (storage, recharge) across various locations and depths in aquifers is not sufficiently described in the literature, which lacks an estimate of the aquifers' sustainable output at various sites and depths. Most groundwater quality investigations with sizable sample sizes are carried out in Kailali, Kanchanpur, and Dang districts, but similar studies are also necessary for other regional districts to properly define the spatial distribution of groundwater quality in the FtF-ZoI region.

As vital as evaluating the existing state of groundwater resources is, understanding the recharge process and prospects for recharge augmentation are equally important to ensure a continued supply of groundwater for the projected CM. It calls for knowledge of recharge trends and seasonality, groundwater storage and availability, and evaluating various strategies for recharge augmentation and water sources protection. Eventhough this information is extremely significant, it is not commonly known because of lack of relevant studies in Nepal or the study area.

#### 4.1.4 Prospects for augmentation

Being a part of a large alluvial aquifer of the Indo-Gangetic plain, the groundwater aquifers are recharged relatively faster in the Terai region of Nepal. This argument is also supported by the fact that less than a quarter of 8.8 BCM average annual groundwater recharge is utilized/extracted in the Terai (Nepal et al., 2021). However, given the heterogeneity of the aquifer system, there is spatio-temporal variability in groundwater availability with prospects, therefore, for augmenting water resource availability through rainwater harvesting and managed aquifer recharge. However, no studies or practical experiences of this have been documented. This means that a detailed scientific study is needed to identify an appropriate location, sizing rainwater harvesting and recharge systems, and workable technologies for that specific geography.

Similarly, inter-basin water transfer for bringing water to water-deficit basins from water-surplus basins is already under progress in the FtF-ZoI region. Under the ‘Bheri-Babi Diversion Project’, the Bheri river is being transferred to irrigate the command area of the Babai irrigation project. Several such types of projects, with multiple purposes, can be conceptualized and planned to address water and energy security issues simultaneously.

### 4.2 Water access and productivity

#### 4.2.1 Total water demand

Designing systems for simultaneous use of surface- and ground-water resources requires the understanding of water needs and their spatio-temporal distribution. To develop and execute conjunctive water usage systems, it is essential to identify water demand hotspots, evaluate the availability of water there, and investigate potential water sources. Based on a thorough analysis of 11 items of literature found and screened using a method described by Pandey et al. (2021), the current state of knowledge about water demand was summarized.

The review found that there had not been a thorough investigation into the estimates of water demand within the FtF-ZoI. A few studies have approximated both domestic and irrigation water demand, but none completely cover the FtF-ZoI. For instance, Pandey et al. (2010) reported irrigation and domestic water demand across the basins, revealing that Babai uses the most water (77% of the water resources available), Mahakali uses the least water (2.6%), and Karnali and Mahakali both have significant potential for new water development projects (SM-4). Information on district profiles released by MoALD (SM-5a) and updated information available in the Irrigation Master Plan (IMP, 2019) and other sources (SM-5b) have helped to calculate irrigation water demand in three Terai districts inside the FtF-ZoI area. Finally, SM-5c summarizes pertinent data for evaluating water consumption, including total agricultural area, area under main cereal crops (such as rice and wheat), and areas under surface irrigation systems. Furthermore, probable groundwater hotspots mapped as distribution of deep tubewells throughout Nepal's Terai districts and published in a study by Pathak (2018) can also be considered as a proxy for spatial distribution of water demand.

#### 4.2.2 Water access – status and constraints

The FtF-ZoI region has a long history of water development activities aimed at improving water access and GoN implements numerous water resource development initiatives and programs in coordination with international aid organizations and academic institutions. Many involve small-scale irrigation, hydropower, sanitation, and water supply, some of which Pandey et al. (2021) summarise with a particular focus on water resources and irrigation. As irrigation accounts for most water consumption, only irrigation-related schemes are included in this article. The Irrigation and Water Resources Management Project (IWRMP), Non-Conventional Irrigation Technology Project (NITP), Medium Irrigation Project (MIP), and Community Managed Irrigation Agriculture Project (CMIASP) are some noteworthy efforts. In essence, NITP supports micro-irrigation technologies and creates effective irrigation systems for them. Table 1 provides an overview of key irrigation development factors in the FtF-ZoI districts (Terai only).

**Table 1:** Irrigation systems and their key features within the FfF-ZoI region (Sources: Pandey et al., 2021).

District	Total cultivated area (ha)*	Total area under irrigation (ha)	Surface irrigation system					GW irrigation system						
			GCA (ha)	NCA (ha)	No. of irrigation system	No. of AMIS	No. of LIS	Total GCA under GW irrigation (ha)	Deep tube well (DTW)		Shallow tube well (STW)		Area under DTW (%)	Area under STW (%)
									No. of wells	GCA (ha)	No. of wells	GCA (ha)		
Kapilbastu	96,255	28,826	24,023	15,915	24	1	3	4803	85	1825	2978	2978	38	62
Dang	119,497	37,911	31,322	27,548	119	0	0	6589	119	2565	4024	4024	39	61
Banke	65,191	48,461	39,457	25,076	30	1	4	9004	82	2565	6439	6439	28	72
Bardia	69,876	72,718	62,978	34,921	14	2	0	9740	35	1080	8660	8660	11	89
Kailali	102,457	76,614	45,195	29,554	23	1	0	31,419	75	2315	10,799	29,104	7	93
Kanchanpur	61,981	45,733	21,485	16,874	21	1	0	24,248	58	2200	8039	22,048	9	91
**Total**	**515,257**	**310,264**	**224,461**	**149,890**	**231**	**6**	**7**	**85,803**	**454**	**12,550**	**40,939**	**73,253**	**15**	**85**

Notes: 1) AMIS: Agency-Managed-Irrigation-System; GCA: gross command area; GW: groundwater; ha: hectare; LIS: lift irrigation system; NCA: net command area; No.: Number; 2) STW and DTW data are based until July 2017. Command areas in the Table are planned and do not include private investment (e.g. in groundwater); therefore, they may not reflect the actual served area. Tubewells with depth < 50 m are considered as STWs. 3) * Cultivated area is taken as agricultural area based on ICIMOD (2010).

Despite availability of water resources and several initiatives, access to irrigation is still a problem, as stated in Section 3. A major part of this is more due to economic water scarcity than physical scarcity. The anticipated shift in hydrology and streamflow brought on by climate change is likely to further affect water availability (Pandey et al., 2020b). The majority of the FtF-ZoI districts have access to both surface- and ground-water, with the latter supplying irrigation to 28% of the total irrigated land. Proper handling of the issue of economic water scarcity by reducing energy costs means that groundwater has strategic importance in the satisfying spatio-temporal distribution of water demand due to its availability in aquifers in the Terai. Please refer to SM-6 for the overall status of access to surface- and ground-water irrigation.

#### 4.2.3 Water use practices – preferences and efficiencies

According to hydrogeological mapping, the Terai has enough renewable groundwater resources to provide effective irrigation of agricultural farmlands (Nepal et al., 2021), with only about 22% of its dynamic groundwater recharge capacity have been used thus far (Shrestha et al., 2018). Although a thorough scientific investigation is needed to map the spatio-temporal distribution of groundwater and safe/sustainable yield in connection to irrigated land and cropping systems, sustainable solutions could be implemented to better manage groundwater resources.

Preferences for using one water source over another also have implications for the success of CM making an understanding people’s preferences over the selection of source, selection of irrigation method (for efficiency), awareness of water-related issues and benefits of CM, and behaviourial factors (such as inclination towards compliance with the rule of law) highly valuable information for assessing the prospects for CM in an area. However, as no such information is available in the existing literature, further research is required to characterize these aspects.

#### 4.2.4 Water productivity – status and constraints

Water productivity for the crop can be defined as crop yield per cubic meter of water consumed (Cai and Rosegrant, 2003). Increasing the water productivity in agriculture reduces the need for additional irrigation water and is a critical response to increasing water scarcity. There are many promising pathways to increase water productivity in developing countries including supplemental irrigation, soil fertility maintenance, deficit irrigation, small-scale affordable management practices for water storage, delivery, and application; modern irrigation technologies (such as pressured systems and drip irrigation); and soil water conservation through zero or minimum tillage (Molden et al., 2007).

There have been several experiments to measure the water productivity for different crop establishment and irrigation methods to increase the water productivity and hence profitability of the rice-wheat crop systems in Nepal (Devkota et al., 2019, Mandal et al., 2009). Devkota et al. (2019) compared direct seeded rice (DSR) with puddled transplanted rice (TPR), which is the traditional method of rice cultivation. Similarly, it compared zero tillage wheat (ZTW) with conventional tillage wheat (CTW). Devkota et al. (2019) concluded that the alternative crop establishment methods for rice and wheat are not only commercially viable options but can also produce similar or higher yields while saving water and labour, reducing costs, increasing net profit and B:C ratio. Experiments by Mandal et al. (2009) found similar results regarding water savings and increase in water productivity for alternative crop establishment methods compared to the traditional crop establishment and irrigation methods. It also concluded that conventional tillage, crop establishment and water management practices in rice (TPR) and wheat (CTW) required a large amount of irrigation water and labour compared to the water-wise and cost-effective alternatives for rice (DSR) and wheat (ZTW) establishment.

### 4.3 Energy access and affordability

#### 4.3.1 Availability of energy sources and status of access

Energy is required to pump water from the groundwater table, meaning that access to energy is of prime concern to introduce groundwater to CM. Depending upon location, different energy sources are accessible, such as grid-based energy, solar-based energy, and diesel-based energy for pumps. A vast majority of users use diesel pumps due to their flexibility at the location, time of interest, and availability for hiring. Solar-based and grid-based energy is also being used for pumping groundwater. However, an unreliable power supply and inadequate rural electrification are the challenges in the area. Moreover, comprehensive data/information with quantitative information on access to different energy sources is unavailable; a knowledge gap therefore exists.

#### 4.3.2 Cost of various sources of energy and affordability

Diesel water pumps dominate the water pumping sectors in Nepal, followed by electric water pumps (Consolidated Management Services Nepal Pvt. Ltd., 2013). The rising cost of diesel and the increasing rental charge of pump sets have consistently been a cause of disappointment among farmers of all categories. Urfels et al. (2020) listed key factors that limit shallow tubewell use in areas with a predominance of diesel pumps. They include poor coordination among water users, delays in pump and tubewell availability, financial constraints, and risk aversion towards cash investment. It was also suggested that the electric grid permits lower-cost pumps, and solar-powered irrigation systems could reduce operating costs. The aversion to cash investments can be overcome at the farm level by raising awareness of the importance of timely irrigation coupled with efforts to increase operational efficiency (e.g., pump maintenance, pump sizing, and forecast-based irrigation scheduling (Urfels et al., 2020)).

The GoN has introduced policies and mechanisms, subsidies and credit financing to encourage renewable energy in the irrigation sector to improve agricultural productivity and production (Renewable World and ACIAR, 2018; Thapa et al., 2020). Solar technology is one of the new technologies able to reach farmers who do not have access to grid power and who are ready to switch from fossil fuel-based to clean energy technologies. Comparative studies of the cost per unit of water in different pumping technologies show that the cost of the electric pump is comparatively the lowest, followed by the solar water pump when capacity utilization factor (CUF) is above 50% (Renewable World and ACIAR, 2018; Thapa et al., 2020). The comparative cost for different pumping technologies is shown in Table 2.

**Table 2:** Variation in cost of water under different pumping technologies (Source: Thapa et al., 2020).

Solar Pump Capacity Utilization Factor (%)	Water Volume (m^3^/day)	Cost (NPR /m^3^ of water)		
		Solar Pump	Diesel Pump	Electric Pump
90	55.40	2.83	5.26	2.90
70	43.09	3.60	5.48	3.06
50	30.78	5.00	5.51	3.14
25	15.39	9.80	6.39	3.81
10	6.16	24.40	8.80	5.58

Note: NPR is Nepali Rupees; 1 NPR = 0.0076 USD (28 February, 2023.

While solar pumping seems an attractive option when used at about 50% CUF, the high upfront cost, inaccessibility to technology and after-sales services are hindering to popularization and flourishing the system among smallholder farmers. The rising cost of diesel and increasing rental charge of pump sets have consistently been a cause of disappointment among farmers of all categories.

### 4.4 Water governance landscape

Khadka et al. (2021) provided a summary of the overall governance environment relating to the development of water resources in the FtF-ZoI. There are restrictions and issues with groundwater-based irrigation due to numerous legislative, institutional, investment, and governance issues (Sugden et al., 2020; Urfels and Foster, 2020). Scaling farmer-led irrigation, as well as CM of surface water and groundwater resources, is systematically hampered by inadequate consideration of the local socio-economic context, the larger enabling environment, and governance aspects of irrigation, as well as by a greater focus on large physical infrastructures and engineering-centered approaches (Khadka et al., 2021). Overcoming those obstacles will need inclusive and sustainable irrigation development and multi-actor participation in irrigation planning, decision-making, and investment (Minh et al., 2020, Khadka et al., 2021).

#### 4.4.1 Policy landscape regarding CM

CM has been emphasized and prioritized in policy documents since the early 1990 s, thus providing ample space for its promotion. However, translating the policy and strategies into action is yet to achieve momentum. Nepal’s Irrigation Policy 1992 mentioned the need for promoting CM to optimize the use and management of available water resources to increase irrigated agriculture; while the Agricultural Perspective Plan 1995 emphasized the use of groundwater and the intensification of shallow tube wells, implying the idea to introducing groundwater use into the system where irrigation was predominantly gained from surface water, thus promoting CM of both resources.

Similarly, activities under the fourth output of Water Resources Strategy 2002 mention developing YRI and improving groundwater development and management, implying prioritization of CM. Policy principles adopted in National Water Plan 2005 mention that water resource development and management shall be undertaken in a holistic and systematic manner, relying on IWRM. It also implies that both surface water and groundwater should be developed and managed in an integrated way, providing space for planned CM. Although it did not spell out CM in the action programs, it focused on expanding groundwater development. Nevertheless, targets for increasing YRI and irrigation efficiency imply a focus on the planned CM. Also, Irrigation Policy 2013 mentions CM in the suggestions bullet of conception (ABADHARANA) section.

Similarly, section 1.6.2 of the Policy mentions considering prospects for CM as one of the criteria for selecting an irrigation project. Furthermore, Strategy-2 and its first Policy/KARYANITI 1 of the Water Resources Policy 2020 highlight on integrated management of water resources. The sixth POLICY/KARYANITI of the seccond Strategy mentions prioritizing CM based on prospects and needs.

#### 4.4.2 Institutional settings (to promote CM)

An enabling environment to facilitate CM can be created by setting up a single institution with the mandate/authority to look after the development and management of both surface- and ground-water resources. However, in the context of this study area in particular and the entire Nepal in general, the authority to manage surface- and ground-water lies with different institutions, thus, creating a barrier to promoting planned CM. Ten sectoral ministries are also involved in water resources, watershed management, and irrigation services and development, even though MoEWRI is the primary line ministry at the federal level setting water resources and irrigation policies (Khadka et al., 2021). If MoEWRI does not create and implement an institutional water cooperation and development system, there is a greater likelihood that strategies and programs will be duplicated at the federal level.

#### 4.4.3 Social and equity aspects of CM

Gender and social equity issues of water access, management and use in the FtF-ZoI are complex and institutional mechanisms, policies and programs of the state and non-state actors must integrate these issues in CM. The GoN has implemented several private irrigation subsidies to assist smallholders and boost technology adoption. However, irrigation technology subsidies mostly benefit better-off farmers due to a lack of inclusive criteria and application processes (Khadka et al., 2021). In addition, although women provide the main source of labour for irrigated agricultural production and value chains, they have limited access to technologies and information regarding multiple-use water services and skills, or knowledge of the impacts of the climate crisis on the water and food systems.

Moreover, irrigation technology and infrastructure are not gender-friendly (Khadka et al., 2021). The implementation of inclusive principles outlined in national policies has been constrained by the persistence of the interest of water professionals and institutions in the technical aspects of irrigation development and the dearth of social science expertise, knowledge, and effort in water intervention. (Shrestha and Clement, 2019). This scenario reveals that Nepal’s water and irrigation sectors need to approach water development differently, and water interventions should focus on empowering smallholder farmers and women in order to capitalize on the potential positive impacts of CM on people's livelihoods, the environment and water security (Khadka et al., 2021). As the Constitution of Nepal 2015 has devolved more roles and responsibility for local water supply, irrigation and watershed conservation to local governments, there is a need for policy and institutional capacity development at the local level to implement gender equitable and socially inclusive water resource management and use (Khadka et al., 2021).

#### 4.4.4 Environmental aspects

CM aims at maximizing water productivity and minimizing environmental externalities. Relevant regulations for limiting groundwater extraction, penalties for over-extraction of groundwater, and guidelines for minimizing groundwater pollution/contamination should therefore be in place to support the implementation of CM. However, these aspects are yet to gain attention in the FtF-ZoI area.

### 4.5 Capacity and awareness

#### 4.5.1 Human resources or technical capacity

A generic understanding of CM exists among stakeholders in the FtF-ZoI. However, detailed technical knowhow on realizing the CM is lacking among farmers and the government’s implementing agencies. It is primarily because CM is neither prioritized in the university curriculum nor in various capacity-building programs organized over time. As skilled human resources in relevant agencies are the key to the successful design and implementation of CM, tailored training programs with a specific focus on its various aspects are certainly essential for stakeholders related to water and irrigation and working in the FtF-ZoI area.

#### 4.5.2 Financial capacity (for investment in CM)

The government’s spending on irrigation is continuing to increase; however, investment is not properly focused. CM does not need significant extra investment but rather strategizing and reorienting current investments better. As revealed by interaction with relevant stakeholders, financial capacity is therefore not a key constraint for CM in the study area.

#### 4.5.3 Understanding (of various stakeholders) and practices of CM

Various types of stakeholder are aware of the CU/CM concept and consider it a good one, but remark on its lack of demonstration. Until the 5th periodic plan (1975–80), the focus was on providing adequate irrigation for paddy; the concept of YRI started to gain attention from the 6th periodic plan (1980–85). This concept then attracted the idea of CM, although it is referred to as CU in these policies and planning documents. For many years, however, Nepal has been unable to present a successful case of CM.

Though CU is in default in many cases, there is no evidence of successful CM cases in Nepal. The Arjun Khola irrigation system located in Deupur in Lamahi, Dang, was designed probably as the first case for CM back in 1994 to irrigate 475 ha of command area. The Arjun Khola had adequate water for the wet season but no water for the dry season, and so three deep and three shallow tubewells were constructed to supplement the dry-season water deficit. Initial results were good, but it failed for various reasons, one being the rigidity of the distribution system, as the conveyance system designed for the surface water system is not appropriate for the groundwater system. For example, in the case of deep tubewells, the field channel sizes required for dry and winter seasons are entirely different. This is because larger conveyance systems in the dry season would result in more losses and costs for water pumped from the groundwater system. Other potential reasons include but are not limited to: i) differential cost for surface water and groundwater (i.e., a greater cost for groundwater use than for surface water use); ii) lack of exposure and therefore capacity in the planning of CM system; iii) unavailability of technical expertise for O&M in the locality; iv) institutional aspect (i.e., the working culture withboth surface- and ground-water personnel enjoying working independently; v) operation and maintenance aspect (e.g., demand charge, potential risk of failure of the system while not working for six months or so during the wet season, etc.); vi) unequal access to groundwater based on gender and socio-economic conditions; and vii) difference in the subsidy/ technical assistance provided by the government for surface- and ground-water.

## 5 Strategies to implement CM in Western Nepal

### 5.1 Challenges and impediments

The development of a CM system faces constraints in the FtF-ZoI areas (Terai) of Nepal. Inadequate technical capabilities and organizational structure, socio-political realities, a lack of emphasis on research-based development approaches, data/information shortages, and a lack of encouraging and disseminating research are some of its underlying causes. Some of the barriers that Foster et al. (2010) outlined also apply to this research area; some of the constraints are.

• limited availability of adequate and reliable data on groundwater availability, annual/seasonal consumption and recharge patterns within the study area; and lack of an inventory of groundwater abstraction wells in the entire study area
• inadequate knowledge of the limits of groundwater development and its spatio-temporal dispersion
• lack of integrated modelling of water systems (both surface- and ground-water), canal supplies, irrigation water needs, and agricultural output to produce empirical evidence of the effects of various combinations and scenarios of CM systems on social and economic consequences for CM planning
• lack of demonstration cases, reliable information, and suitable communication product(s) on CM communication opportunities/prospects with various stakeholders; the spatio-temporal advantages of CM vs. nothing under various water and food security situations must be determined
• only sporadic scientific studies exist on water availability assessment (e.g., Pandey et al., 2019, Pandey et al., 2020b; Dahal et al., 2020), inundation modelling and flood risk assessment, prioritizing river basins for flood risk management (DWRI/WRPPF, 2020a, DWRI/WRPPF, 2020b, DWRI/WRPPF, 2020c); studies that consider water withdrawal patterns for agricultural applications and the maintenance of environmental flows are scarce. Similar to how groundwater irrigation command areas are referenced in literature, there is no information about CM of surface- and ground-water across the study area. There is a void in the literature about the current state of CM, its limits, lessons learned, and empirical data supporting its widespread promotion; such a discrepancy in focus and information availability is brought on by inadequate financial allocation emphasis on research for development projects
• as Foster et al. (2010) stated, the frequent division of labour between surface- and ground-water production and/or management frequently makes it difficult to recognize and engineer opportunities for planned CM; it is vital therefore to have a single entity with the mandate and power to manage both surface- and ground-water resources.
• lack of understanding of the importance of moving beyond an operationally easy self-supply system to a more secure and resilient CM solution due to various factors, including inadequate instruction in academic courses
• an unreliable power supply and insufficient rural electrification for groundwater pumping in canal command regions; this can however be overcome using well-designed solar irrigation pump programs which provide universal access to energy.
• under -developed institutional and policy ability at the local level, needed to undertake management and use of water resources which are gender-equitable and socially inclusive
• the great majority of pumps currently used to power irrigation in the study districts (Terai) are run on diesel which contributes to climate crisis; however, these can be replaced with solar irrigation pumps

### 5.2 Potential strategies to realize the prospects of CM

Strategies, resources, and commitments to overcoming these hurdles (Section 5.1 ) must be implemented, to turn the prospect of CM in the study districts (Terai) in Western Nepal into a reality. The following subsections focus on appropriate tactics as well as areas of investment identified for the study districts in the Terai.

#### 5.2.1 Realign institutional mechanisms

Existing policies, plans and strategies adequately emphasize CM; however, Nepal’s institutional mechanisms have not been realigned in line with their spirit in order to put it into effect. An enabling environment for promoting CM can be created by realigning institutional mechanisms where surface- and ground-water in a command area and/or basin are managed and developed through a single entity such as a river basin authority. A starting point could be piloting this concept in a particular catchment and scaling it up based on the learnings. The main concept is to encourage integrated use and management of surface- and ground-water resources while considering command areas as sub-units and **river basins as units**. Removing the fragmented structure of governmental institutions (including water management institutions) charged with various water management functions may be necessary to effect reform on several fronts. To attract the private sector to the anticipated CM irrigation schemes, institutions must create an environment conducive to public-private partnerships and community-based investment. Engaging the private sector in cost recovery, particularly in operation and maintenance, may be difficult; however, institutions can develop an appropriate model through a few pilot projects in various contexts to ensure more return with less expenditure of public funds. To achieve gender equitable and socially inclusive water resource management and use, the institution should also support policy and institutional capacity development at the local level. They can then work to improve energy and water use while also advocating for a reliable power supply in the command areas to support CM. This can be done by encouraging solar irrigation programs in areas where grid connection has not yet been reached or there is an unreliable power supply.

#### 5.2.2 Strengthen data assets

Prioritizing **investments** will help improve the **monitoring** of both surface- and ground-water resources. The initial strategic interventions needed to achieve the intended CM project are appropriate monitoring (both quantity and quality) and use, as well as building-related mechanisms/systems for database maintenance, analysis, and dissemination/access. A geographic database containing numerous factors, including cropping patterns, evapotranspiration/water demands, groundwater levels, canal alignments, and groundwater abstraction, may be included in the routine monitoring. In the end, the monitoring will help determine which investments should be prioritized for hardware (system modernization and improved infrastructure), software (improved database), planning and management capabilities, and institutional reforms. In addition, it will offer insights into designing interventions to increase water use efficiency through various paths, such as crop diversity to control water demand, to reduce irrigation system losses, and encourage micro-irrigation systems where suitable. In addition, initiatives for improving recharge and spring source protection must be designed and implemented to support CM.

#### 5.2.3 Research, awareness and capacity strengthening

Investments in ‘research for development’ initiatives are advised to prioritize CM interventions according to the available data. With changes in levels of groundwater usage in space and time and in surface water distribution, this also incorporates integrated numerical modelling of irrigation canal flows, groundwater use and aquifer response, and soil-water status. Several models have been proposed and used to maximize the benefits of water use (e.g., Paudyal and Gupta, 1990; Onta et al., 1991). However, an integrated modelling approach, such as that proposed by Foster et al. (2010), would be more suitable, as it simultaneously integrates groundwater flow processes, runoff, canal inflows, crop water requirements and crop yield. As sustainable development and water resource management involve awareness of biophysical and socio-institutional components, research on the social and institutional aspects of CM and its translation into development are equally vital. Ultimately, this would increase CM efficiency, sustainability, and gender-equitable and socially-inclusive access to and use of water resources. In addition, long-term assistance for the agricultural economy can come from research on sustainable agriculture, water use efficiency, soil erosion control, water quality/quantity restoration, and environmental risk assessment. Furthermore, a major problem that needs to be solved through suitable study is how to translate legislative provisions on CM into actual practices and activities.

It is advised to conduct a thorough investigation into the Terai aquifer, a portion of the Gangetic Plain, with a focus on integrated modelling of the whole surface and groundwater system, including the Chure region, and evaluate the effects of various types of interventions on Terai's groundwater system. In the case of hills, studies targeting defining recharge regions and evaluating the efficacy of various types of interventions in these recharge areas in recharge source/area protection would be a good method to ensure groundwater supply for sustaining CM. Evidence for these interventions must be derived from monitoring data and subsequent analysis.

Finally, evidence-based communication materials must be created to launch a long-term campaign for raising awareness of effective water use, conservation of available water sources, and education focusing on various risks and costs associated with water mismanagement, as well as the advantages of CM and methods for its planning and implementation.

## 6 Conclusions and recommendations

Access to irrigation is considered a primary constraint for optimal and stable crop yield, and therefore a threat to food security in Nepal. The CM of surface- and ground-water can be considered an adaptation strategy. However, an integrated framework for assessing the prospects for CM and its implementation is lacking in the literature. In this context, this study developed a five-component and 17- indicator framework for CM through literature review, stakeholder consultation and expert interviews as elaborated in section-2. This was then applied to synthesize the overall prospects of CM in Nepal’s Western Terai, first by providing the context of the region’s importance in national food security (section-3) and then discussing the status of each indicator (section-4). Finally, potential strategies for realizing these prospects were highlighted along with their implementation challenges (section-5). Key conclusions are highlighted below.

Water resource availability is relatively abundant. The understanding of surfacewater availability and its spatio-temporal distribution is good. For example, with surfacewater, information is available for nine basins/sub-basins in the area, albeit with varying levels of detail, on essential hydrological features (such as catchment area, amount of water and water balance components). Specific discharge varies from 18.8 l/s/m^2^ (Upper Karnali) to 52.1 l/s/m^2^ (Mohana) across the river systems. However, an integrated framework that looks at the overall water availability holistically across the entire region, an adequate monitoring framework for both surface- and ground-water resources, and prospects for augmenting water availability and developing water resources is lacking. An integrated modelling of the entire hydrological system (and its regular updating) is crucial because adequate and integrated CM and water resources planning will not be achievable without tools, processes, and data that drive scenario development to support policy prioritizing and decision-making.

Despite availability, access and productivity of water resources is low for various reasons such as lack of focus on estimating and monitoring water availability and use, inadequate efforts to understand water use practices and preferences; and low prioritization of systematic and continuous efforts towards enhancing water productivity. Energy is a key input for enhancing access to water in the context of Western Terai. Given the flat nature of terrain, its access is relatively good, with availability of different sources of energy. However, cost and affordability is an issue in some instances, particularly for smallholder farmers.

Water governance landscape, capacity and awareness are key to creating an enabling environment for promoting CM in an area of interest. Overall assessment of the water governance landscape reveals that the policy landscape is conducive for promoting CM in the Southern Plain region of Western Nepal. However, emphasis on translating the policy into an action plan, setting up an appropriate institutional framework, internalizing the importance of social and equity aspects of CM, and raising awareness of ensuring environmental health while optimizing agricultural productivity, still need a significant attention. One reason for this could be the lack of literature on CM and of demonstration projects in Nepal. In addition, awareness, capacity gaps (basically, re-orienting already competent human resources towards CM), focused/concentrated investments, and regular engagement with stakeholders to understand their concerns and share learnings are equally important aspects for achieving the goal of CM. In the context of the study region, however, these are yet to be prioritized.

Adequate prospects exist for CM to enable year-round-irrigation in the FtF-ZoI districts (the Terai) due to the co-existence of surface water and groundwater, underutilization of groundwater resources, and unreliable or insufficient water supply from surface water systems which draw water from rain-fed and spring-fed rivers. Although challenges and impediments exist on various fronts, three strategies are recommended to translate the prospects for CM into implementation: (1) re-aligning institutional mechanisms in such a way that both surface- and ground-water are dealt with together by a single unit/entity, (2) strengthening data assets through regular monitoring and evaluation of the data, and (3) investment in research, awareness and capacity strengthening programs.

## Declaration of Competing Interest

The authors declare that they have no known competing financial interests or personal relationships that could have appeared to influence the work reported in this paper.

## Data Availability

No data was used for the research described in the article.
