# Supplementary material for: Implementing conjunctive management of water resources for irrigation development: A framework applied to the Southern Plain of Western Nepal
Source: Agric Water Manag. 2023 Jun 1;283:108287. doi: 10.1016/j.agwat.2023.108287 (PMC10167539; doi:10.1016/j.agwat.2023.108287)
Supplement: Supplementary file 1 — Supplementary material [file mmc1.docx]

**Supplementary Materials**

**SM-1: Summary of CM status in FtF-ZoI, Western Nepal**

| Component | Indicator | Status |
| --- | --- | --- |
| 1. Water resources availability and its understanding (current and future) | 1.1 Surface water availability | Five river systems and many southern rivers provide surface water in the FtF-ZoI area. The understanding of surface water availability and its spatio-temporal distribution is good. For example, with surface water, information is available for nine basins/sub-basins in the area, albeit with varying levels of detail, on essential hydrological features (such as catchment area, amount of water and water balance components). Specific discharge varies from 18.8 l/s/m^2^ (Upper Karnali) to 52.1 l/s/m^2^ (Mohana) across the river systems. |
|  | 1.2 Groundwater availability | Except Dang, all Terai districts within the FtF-ZoI have flat topography and have groundwater resources. There are both shallow (0-46 m depth) and deep (> 46m) aquifer systems. Maximum aquifer yields from those aquifers vary from 167 to 2,592 m^3^/day. Model-based analysis revels renewable groundwater availability (m/yr) across the FtF-ZoI districts vary from 0.11 (Dang) to 0.48 (Kanchanpur). Dang’s shallow aquifer alone has an estimated 130-140 MCM/yr of groundwater availability. Though there are estimates of groundwater storage and yield, their updating and mapping their spatial distribution (in high resolution) is lacking, but they are important for CM planning. |
|  | 1.3 Monitoring and scientific understanding of water availability | Monitoring of surface water resources in the medium to large rivers are relatively good, but those at southern rivers are inadequate (or even non-exist in some cases). Furthermore, density of wells for groundwater level monitoring is inadequate. In this context, real-time monitoring system with appropriate mechanism for data collection, storage, and dissemination is yet to be prioritized in the region. Furthermore, an integrated modelling of the entire hydrological system (and its regular updating), which is crucial for CM planning, also demands need for such monitoring system. |
|  | 1.4 Prospects for augmentation | Being a part of a large alluvial aquifer of the Indo-Gangetic plain, the groundwater aquifers are recharged relatively faster in the Terai region of Nepal. Given the heterogeneity of the aquifer system, there is spatio-temporal variability in groundwater availability with prospects, therefore, for augmenting water resource availability through rainwater harvesting and managed aquifer recharge. However, no studies or practical experiences of this have been documented. This means that a detailed scientific study is needed to identify an appropriate location, sizing rainwater harvesting and recharge systems, and workable technologies for that specific geography. |
| 2. Water access and productivity (current and future) | 2.1 Total water demand | A few studies have approximated both domestic and irrigation water demand, but none completely cover the FtF-ZoI. Though there are various information (scattered in different documents) that are useful in estimating water demand, however, a thorough investigation into the estimates of water demand within the FtF-ZoI, mapping hotspots for surface- and ground-water demands are yet to be made. |
|  | 2.2 Water access | There have been several initiatives (of various types and sizes) aimed at enhancing water access, however, despite adequate availability of water resources and several initiatives, access to irrigation is still a problem, primarily due to economic water scarcity. |
|  | 2.3 Water use practices | Though a thorough scientific investigation is needed to map the spatio-temporal distribution of groundwater availability and its safe yield, only 22% of the Terai’s dynamic recharge capacity has been utilized so far. Valuable information required for assessing water use practices such as preferences over the selection of source, selection of irrigation method, awareness of water-related issues and benefits of CM, and behavioural factors, which are required for assessing the prospects of CM, are not available so far. |
|  | 2.4 Water productivity | There are sporadic studies on evaluating water productivity in the FtF-ZoI region, however, a comprehensive information on water productivity in the region and its variation with various factors are not well understood. |
| 3. Energy access and affordability | 3.1 Available energy sources and status of access | Depending upon location, different energy sources are accessible in the FtF-ZoI region. They include grid-based energy, solar-based energy, and diesel-based energy for pumps. A vast majority of users use diesel pumps. comprehensive data/information with quantitative information on access to different energy sources is unavailable |
|  | 3.2 Cost of various sources of energy and affordability | The rising cost of diesel and the increasing rental charge of pump sets have consistently been a cause of disappointment among farmers of all categories. Comparative studies of the cost per unit of water in different pumping technologies show that the cost of the electric pump is comparatively the lowest, followed by the solar water pump when capacity utilization factor (CUF) is above 50%. |
| 4. Water governance landscape | 4.1 Policy landscape in regard to CM | CM has been emphasized and prioritized in policy documents since the early 1990s, thus providing ample space for its promotion. However, translating the policy and strategies into action is yet to achieve momentum. Agricultural Perspective Plan 1995, Water Resources Strategy 2002, National Water Plan 2005, Irrigation Policy 2013, and Water Resources Policy 2020 are example of policies and strategies that provide space for CM. |
|  | 4.2 Institutional settings (to promote CM) | The authority to manage surface- and ground-water lies with different institutions, thus, creating a barrier to promoting planned CM. Ten sectoral ministries are also involved in water resources, watershed management, and irrigation services and development, even though MoEWRI is the primary line ministry at the federal level setting water resources and irrigation policies. |
|  | 4.3 Social and equity aspects of CM | Gender and social equity issues of water access, management and use in the FtF-ZoI are complex and institutional mechanisms, policies and programs of the state and non-state actors must integrate these issues in CM. Although women provide the main source of labour for irrigated agricultural production and value chains, they have limited access to technologies and information regarding multiple-use water services and skills, or knowledge of the impacts of the climate crisis on the water and food systems. |
|  | 4.4 Environmental aspects | Relevant regulations for limiting groundwater extraction, penalties for over-extraction of groundwater, and guidelines for minimizing groundwater pollution/contamination should therefore be in place to support the implementation of CM. However, these aspects are yet to gain attention in the FtF-ZoI area. |
| 5. Capacity and awareness | 5.1 Human resources or technical capacity | A generic understanding of CM exists among stakeholders in the FtF-ZoI. However, detailed technical knowhow on realizing the CM is lacking among farmers and the government’s implementing agencies. Tailored training programs with a specific focus on its various aspects are certainly essential for stakeholders related to water and irrigation and working in the FtF-ZoI area. |
|  | 5.2 Financial capacity (for investment in CM) | Financial capacity is therefore not a key constraint for CM in the study area. The government’s spending on irrigation is continuing to increase; however, investment is not properly focused. CM does not need significant extra investment but rather strategizing and reorienting current investments better. |
|  | 5.3 Understanding (of various stakeholders) and practices of CM | Various types of stakeholder are aware of the CU/CM concept and consider it a good one, but remark on its lack of demonstration. Until the 5th periodic plan (1975-80), the focus was on providing adequate irrigation for paddy; the concept of YRI started to gain attention from the 6th periodic plan (1980-85). This concept then attracted the idea of CM, although it is referred to as CU in these policies and planning documents. For many years, however, Nepal has been unable to present a successful case of CM. |

**SM-2(a)**: Surface water availability in the river systems in the FtF-ZoI

| S.N. | Basin name [Outlet] | Basin area (km^2^) | Baseline period | Baseline discharge (m^3^/s) | Baseline water availability (MCM/yr) | Change in water availability in future time period | References |
| --- | --- | --- | --- | --- | --- | --- | --- |
| 1 | Chamelia [Confluence with Mahakali] | 1,603 | 2001-2013 | 66.0 | 2,081 | NF (2021-2045): +8.2% (RCP4.5); MF (2046-2070): +12.2% (RCP4.5);  FF (2071-2099): +15.0% (RCP4.5) | Pandey et al. (2019) |
| 2 | Mahakali [Outlet] | 5,410 | - | 247 (from Nepal) | 7,789 | No data available | WECS (2011) |
| 3 | Mohana [Confluence with Karnali] | 2,918 | 1980-2005 | 152 | 4,793 | NF (2018-2040): 16% (RCP4.5), 24% (RCP8.5); MF (2041-2070): 4% (RCP4.5), 14% (RCP8.5); FF (2071-2099): 17% (RCP4.5), 18% (RCP8.5) | Tripathee (2018) |
| 4 | Karnali [Chisapani] | 42,890 | 1995-2009 | 1,414.3 | 44,602 | NF (2021-2045): 0.6% (RCP4.5), 9% (RCP8.5); MF (2046-2070): 6.4% (RCP4.5), 4.2 (RCP8.5); FF (2071-2095): 8.4% (RCP4.5), 10.9% (RCP8.5) | Pandey et al. (2020) |
| 5 | Karnali [Chisapani hydrological station] | 42,457 | 1981-2005 | 1,375 | 43,362 | MF (2040-2069): 6.4% (5.1%) for RCP4.5 (RCP8.5); FF (2070-2099): 8.4% (10.9%) for RCP4.5 (RCP8.5) | Dahal et al. (2020) |
| 6 | Karnali [near Chisapani hydrological station] | 42,845 | 1985-2005 | 1,480 | 46,673 | No data available | Dhami et al. (2020) |
| 7 | Karnali [Chisapani] | 45,269 | 1981-2010 | 1,369 | 43,173 | No data available | Khatiwada (2016) |
| 8 | Karnali [above confluence with the Ganges in India] | 63,700 | - | 1,900 | 60,000 (approx.) | No data available | IMP (2019) |
| 9 | Karnali-Mohana [Nepal-India border] | 49,892 | 1995-2009 | 1,467 | 46,250 | No data available | Pandey et al. (2020) |
| 10 | Bheri [Jammu Hydrological Station] | 12,290 | 1995-2009 | 361 | 11,383 | NF (2021-2045): -5.4% (RCP4.5), -2.5 (RCP8.5); MF (2046-2070): +3.0% (RCP4.5), +1.3 (RCP8.5); FF (2071-2095): -0.5% (RCP4.5), -3.1% (RCP8.5) | Pandey et al. (2020) |
| 11 | Bheri [Jammu hydrological station] | 13,900 | 1975-2005 | 415 | 13,087 | NF (2020-2044): +7.1% (RCP4.5), +6.0% (RCP8.5); MF (2045-2069): +6.2% (RCP4.5), +7.2% (RCP8.5); FF (2070-2099): +7.3% (RCP4.5), +12.5 (RCP8.5) | Mishra et al. (2019) |
| 12 | West Seti [above Bangna Hydrological station] | 7,460 | 1995-2009 | 283.6 | 8,944 | NF (2021-2045): +13.9% (RCP4.5), +14.5 (RCP8.5); MF (2046-2070): +13.8% (RCP4.5), +16.0 (RCP8.5); FF (2071-2095): +16.1% (RCP4.5), +13.2% (RCP8.5) | Pandey et al. (2020) |
| 13 | West Seti [above GhopaghatHydrological station] | 4,324 | 1996-2005 | - | - | NF (2020-2044): +16.4%; MF (2045-2069): +18.3%; FF (2070-2099): +19.9% (all projections are under RCP8.5 scenarios) | Bhatta et al. (2020) |
| 14 | Tila [above Nagma hydrological station) | 1,870 | 1995-2009 | 46.6 | 1,470 | NF (2021-2045): -21.6% (RCP4.5), -19.5% (RCP8.5); MF (2046-2070): -1.2% (RCP4.5), -17.2 (RCP8.5); FF (2071-2095): -13.4% (RCP4.5), -13.2% (RCP8.5) | Pandey et al. (2020) |
| 15 | Upper Karnali [above Lalighat hydrological station | 15,200 | 1995-2009 | 285.2 | 8,993 | NF (2021-2045): -7.3% (RCP4.5), -7.2% (RCP8.5); MF (2046-2070): +2.3% (RCP4.5), -0.3% (RCP8.5); FF (2071-2095): -1.0% (RCP4.5), -5.8% (RCP8.5) | Pandey et al. (2020) |
| 16 | Babai [above Nepal-India border] | 3,000 | - | 88.8 | 2,800 | No data available | Pandey et al. (2010) |
| 17 | WECS [above the confluence with Karnali river at about 50 km downstream of border] | 3,400 | - | 103 | 3,248 | No data available | WECS (2011) |
| 18 | West Rapti [above Nepal-India border] | 3,380 | until 2010 | 101.5 | 3,200 | No data available | Pandey et al. (2010) |
| 19 | West Rapti [above Jalkundi hydro-station] | - | 1982-2005 | - | - | FF (2071-2100): Increment range from .6 m^3^/s to 8.1 m^3^/s for RCP 4.5 and from 8.8 m3/s to 44.8 m^3^/s for RCP 8.5. | Shrestha (2017) |
| 20 | West Rapti [above the confluence with Karnali river at downstream of Nepal-India border] | 6,500 | Until 2003 | 224 | 7,064 | No data available | WECS (2003) |

**Notes**: NF is near future, MF is mid-future, FF is far-future, RCP is representative concentration pathway; MCM is million-cubic-meters.

***SM-2(b): Key hydrological features of the river basins/sub-basins in the FtF-ZoI***

| River basin/ sub-basin in the Western Nepal | Catchment area (km^2^) | Average annual rainfall (mm) | Average annual discharge (m^3^/s) | Average discharge of monsoon season (JJAS, m^3^/s) | Specific discharge (l/s/m^2^) | Average annual flow volume (MCM) | Runoff  (mm) | Runoff coefficient  (-) | Average annual evaporation (mm) | Reference |
| --- | --- | --- | --- | --- | --- | --- | --- | --- | --- | --- |
| Karnali | 42,845 | 1,481 | 1,480 | 3,057 | 34.5 | 46,673 | 1,089 | 0.74 | 374 | Dhami et al. (2018) |
| Bheri | 12,290 | 1,202 | 361 | 766 | 29.4 | 11,383 | 926 | 0.77 | n/a | Pandey et al. (2020, b) |
| Seti | 7,460 | 1,921 | 284 | 623 | 38.0 | 8,944 | 1,199 | 0.62 | n/a | Pandey et al. (2020, b) |
| Tila | 1,870 | n/a | 47 | 88 | 24.9 | 1,470 | 786 | n/a | n/a | Pandey et al. (2020, b) |
| Upper Karnali (above Lalighat hydrological station) | 15,200 | n/a | 285 | 580 | 18.8 | 8,993 | 592 | n/a | n/a | Pandey et al. (2020, b) |
| Mohana | 2,918 | 2,010 | 152 | n/a | 52.1 | 4,793 | 1,642 | 0.82 | 747 | Tripathee (2018) |
| Chamelia | 1,603 | 2,469 | 66 | 983 | 41.2 | 2,081 | 1,298 | 0.53 | 381 | Pandey et al. (2019) |
| West Rapti | 3,380 | 1,500 | 101 | n/a | 29.9 | 3,200 | 947 | 0.63 | n/a | Pandey et al. (2010) |
| Babai | 3,000 |  | 98 | n/a | 32.7 | 2,800 | 33 | n/a | n/a | Pandey et al. (2010) |

**Notes**: n/a is not available; mm is millimeters; JJAS is June-July-August-September; MCM is million-cubic-meters.

***SM-3: Groundwater Aquifers and Groundwater Quality***

**Aquifer characteristics**: According to GDC (1994), there are primarily two types of aquifer systems in Nepal's Terai: shallow aquifers (0-46 m depth) and deep aquifers (> 46 m depth) (1994). In Table S-3-1, a schematic of the aquifer system in Nepal's Terai is illustrated, and in Table S-3-2, that of the mid-western and western regions, where FtF-ZoI is located. Sporadic studies discuss aquifer systems and their hydrogeologic characteristics, although those qualities are not thoroughly characterized. For instance, the transmissivities of both shallow and deep aquifers range from 18 to 6,670 m^2^/day for shallow aquifers (0 to 46 m depth) and from 30 to 6,670 m^2^/day for deep aquifers (> 46 m depth) in all six Terai districts of FtF-ZoI. (GDC, 1994). The maximum aquifer yields and hydraulic conductivity at selected wells in Dang, respectively, vary from 167 to 2,592 m^3^/day and 1.3 to 370 m/day (Table S-3-3).

**Groundwater abstraction/yield**: In the Terai and Inner Terai as a whole, Pathak (2018) assessed the current state of groundwater extraction in each district and reported on the status of deep wells. It demonstrated that although wells in other districts utilise aquifers from relatively shallower depths, those in Dang have been using the deepest aquifers (300 m deep) for greater quality and quantity of groundwater. Kanchanpur comes in second with a 28 lps well yield, followed by Bardia district (32 lps), and Dang has a relatively lower yield (only 15 lps). A strong correlation between well depth and average yield suggests that deeper wells have a greater chance of accessing multiple aquifers and the permeable confined aquifer, leading to higher yields than shallower ones. The lowest yield is found in wells between 50 and 75 meters, while the highest yield is found in wells between 175 and 200 meters.

**Table** **S-3-1**: Schematic section through main terai aquifers in Nepal (Adapted from: GDC, 1994). ISTW is shallow tube well; WL is water level; DTW is deep tubewell;.

**
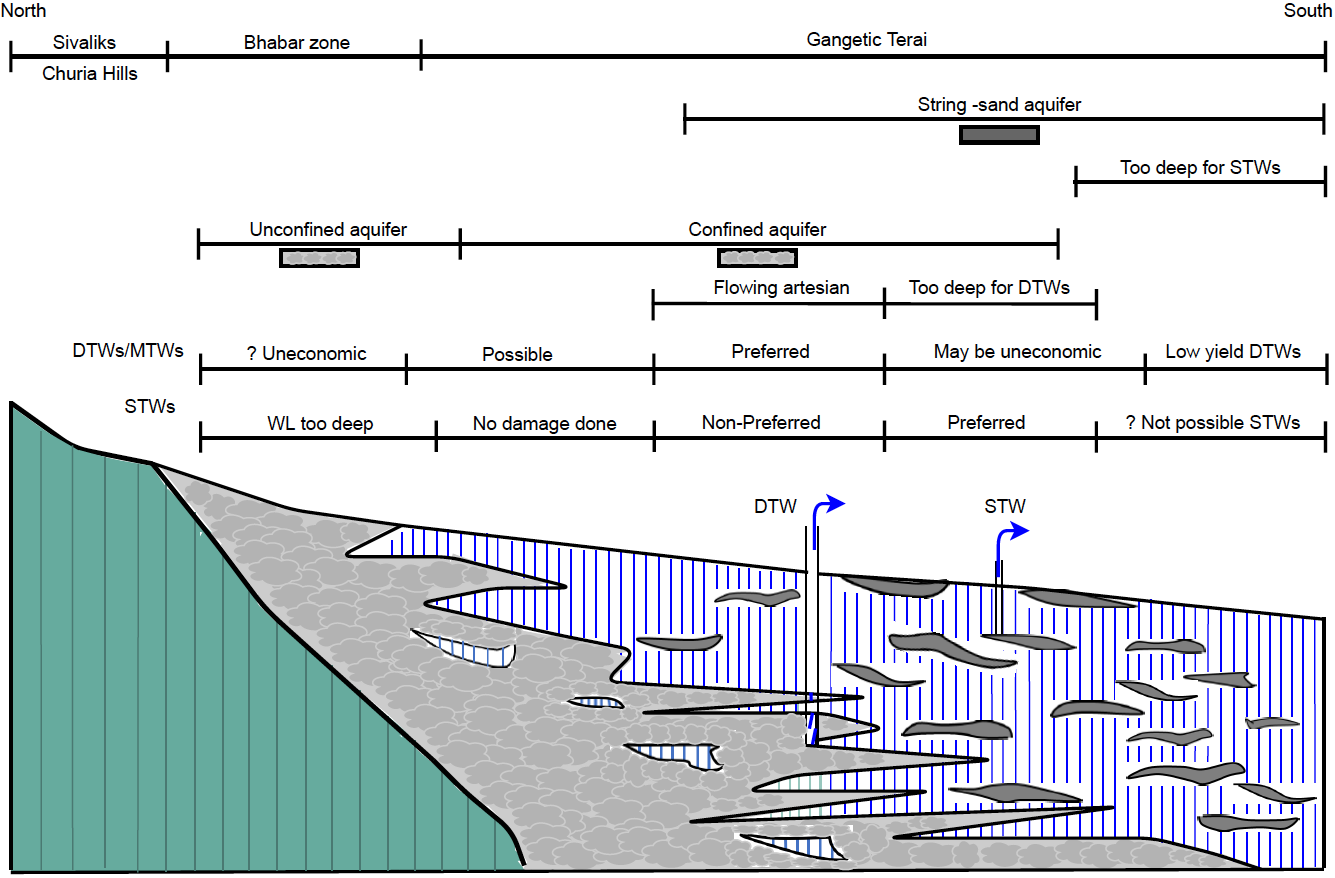
**

**Table S-3-2**: Geological cross-section of – a) mid-western, and b) western (Lumbini area) regions of Nepal (Source: Sharma, 1974)

**
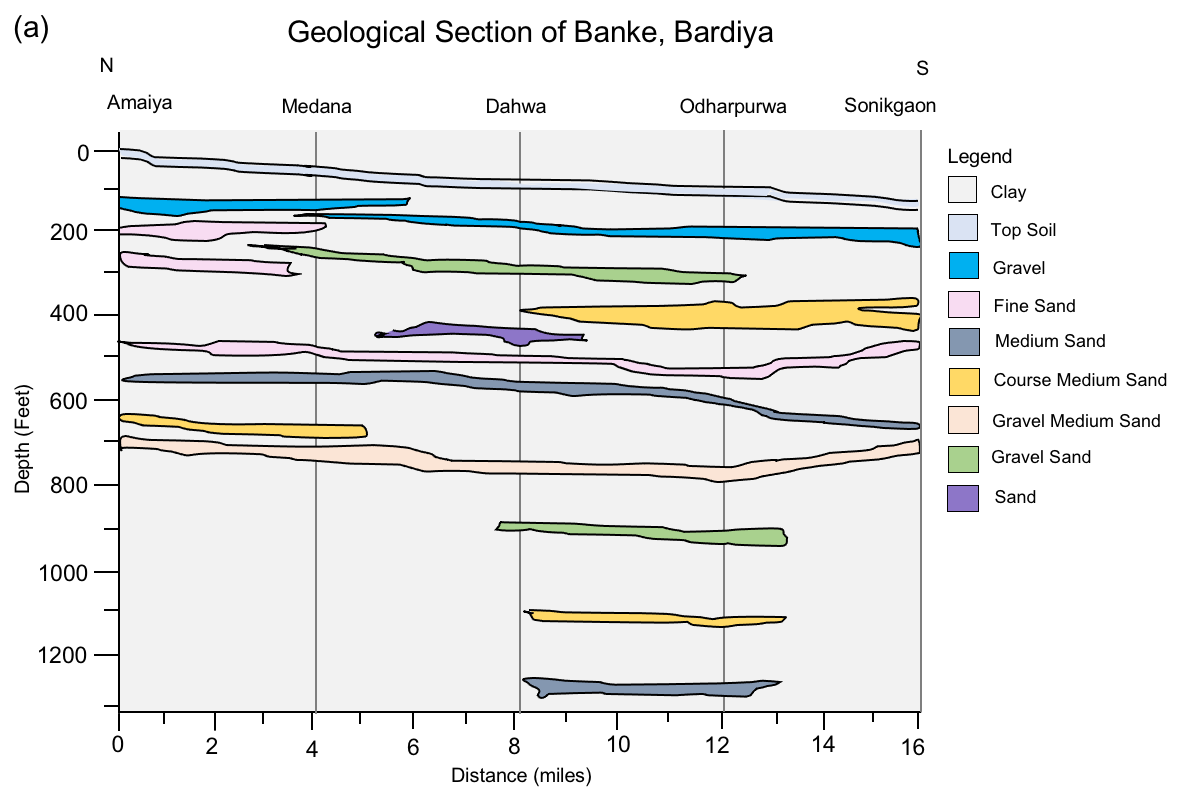
**


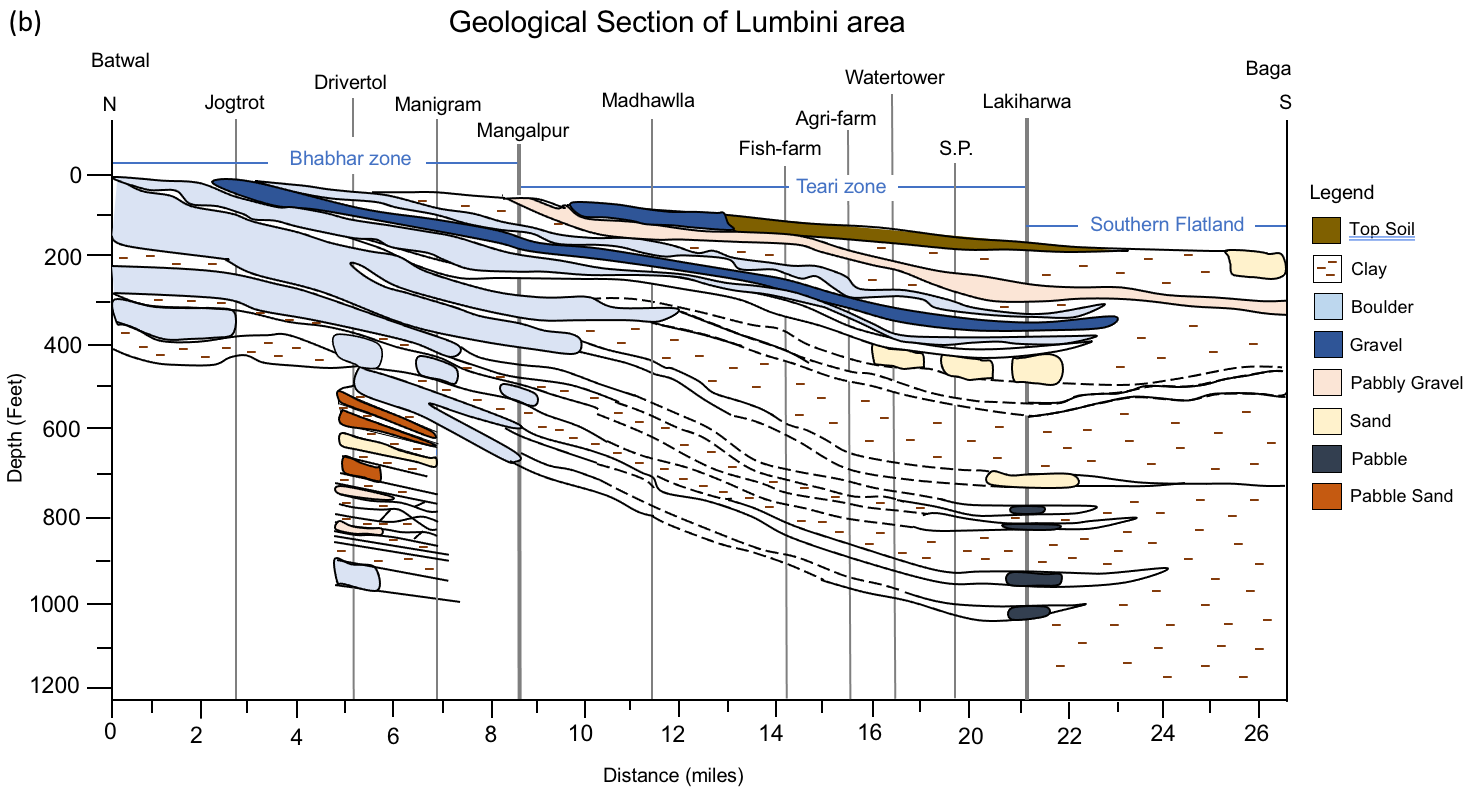


**Table S-3-3**: Hydrogeological characteristics of deep and shallow aquifers in Dang measured in sample observation wells (Source: GWRDB, 1996)

| Well No. | Water Level (masl) | Depth of well (m) | Total cumulative thickness of aquifer (m) | Piezometric surface (m bgl) | Discharge/ maximum yield (m^3^/day) | Transmissivity (m^2^/day) | Hydraulic conductivity (m/day) |
| --- | --- | --- | --- | --- | --- | --- | --- |
| DG/DTW-6 | 632.0 | 70.1 | 9.18 | 15.8 | 1483.5 | 3394.0 | 369.7 |
| TG-5 | 608.0 | 107.0 | 30.0 | 21.0 | 691.2 | 632.5 | 21.1 |
| DG/DTW-9 | 633.0 | 113.5 | 37.2 | 6.0 | - | - | - |
| DG/DTW-21 | 618.0 | 74.4 | 16.5 | 37.5 | - | - | - |
| DG/DTW-7 | 580.0 | 111.2 | 21.4 | - | 630.0 | - | - |
| DG/DTW-3 | 583.0 | 106.1 | - | 11.0 | - | - | - |
| DG/STW-7 | 638.0 | 20.1 | 7.1 | 3.2 | 950.4 | 3477.5 | - |
| DG/DTW-5 | 610.0 | 140.0 | 22.0 | 9.2 | 167.1 | 101.9 | 4.6 |
| NISP/STW-7 | 636.0 | 36.0 | 6.1 | 0.7 | 661.8 | 712.5 | 117.3 |
| DG/STW-6 | 580.0 | 37.5 | 6.1 | 5.0 | 1987.2 | - | - |
| DG/DTW-27 | 586.0 | 111.2 | 33.9 | 5.1 | 2592.0 | 3953.0 | 116.4 |
| DG/DTW-1 | 619.0 | 149.4 | 11.0 | 15.2 | 194.4 | 14.2 | 1.3 |
| DG/DTW-2 | 641.0 | 68.9 | 11.1 | 23.7 | - | - | - |
| Saibahini | 666.0 | 29.0 | - | - | 0.1 | - | - |
| TG-2 | 604.0 | 105.0 | 30.4 | 28.9 | 1036.2 | 2709.3 | 89.1 |

*Notes: masl is meters above mean sea level; mbgl is meters below ground level*

**Groundwater quality**:

Contaminants from geogenic and anthropogenic sources, including arsenic, iron, and microbial diseases, can easily contaminate groundwater. These contaminations can seriously harm the quality of crops and people's health. Few studies describe the FtF-whole ZoI's groundwater quality. In Kailali district, followed by the Kanchapur district, are the areas where studies on groundwater quality are concentrated.

According to Shrestha et al. (2018), the Terai districts of FtF-ZoI have high concentrations of arsenic and other water quality indices (**Table S-3-4**). In the Kailali and Bardia districts, 20% of deep tube wells have arsenic concentrations above 10 ppb. In the other five districts, 4–15% of tube wells had arsenic concentrations above 10 ppb. In 11% of the Tubewells (TW) in the Terai FtF district's Kailali district, the maximum percent of arsenic content was determined to be above 50 ppb.

In six Terai districts in Nepal, including Kailali and Kanchanpur in FtF-ZoI, Yadav et al. (2012) analyzed the susceptibility of arsenic pollution. Out of the six study districts, the Kailali district has the highest mean arsenic concentration (6270 ppb), followed by Kanchanpur (4980 ppb). Arsenic levels gradually rise in shallow tubewells up to a depth of 50 meters before starting to decline below that point. It suggests that drawing groundwater from deeper levels could help prevent the ingestion of arsenic while using groundwater.

Mahat and Shrestha (2008) evaluated the metal and arsenic contamination levels in the groundwater of the Dang district. A total of 523 water samples from tubewells and dug wells were analyzed. The results showed that 10.7 percent and 50.3 percent of the samples contained arsenic above Nepal Standard (50 ppb) and WHO guideline values (i. e., 10 ppb) for drinking water, respectively. In the Dang district's Dhikpur, arsenic levels as high as 240 ppb were discovered. Iron and manganese are both present in significant concentrations (up to 11 mg/l and 0.51 mg/l, respectively).

In the Bhim Datta Municipality of the Kanchanpur district, Bohara (2015) investigated several groundwater sources' physiochemical and microbiological quality. Results indicated that physico-chemical parameters are within the WHO guideline and the national norm. However, the levels of ammonia, chloride, nitrate and total hardness of arsenic were not sufficient. More contaminated than piped or tap water, hand pump and tube well water frequently contained iron.

**Table S-3-4:** Status of water quality in shallow groundwater across the FtF-ZoI. (Source: adapted from Shrestha et al., 2018)

| Parameter | Unit | Districts | | | | NDWS* |
| --- | --- | --- | --- | --- | --- | --- |
|  |  | Banke | Bardiya | Kailali | Kanchanpur |  |
| Ammonia | mg/ L | <0.01-0.38 | <0.01-0.37 | <0.01-0.648 | 0.112– 1.688 | 1.5 |
| Chloride | mg/ L | 12.8– 134.6 | 10.9– 112 | 9.62– 64.1 | 5.128– 41.024 | 250 |
| Electric conductivity | µs/ cm | 420– 1540 | 300– 900 | 150– 640 | 210–710 | 1500 |
| Iron | mg/ L | 0.04– 2.96 | 0.03– 2.21 | 0.03– 3.39 | <0.001-0.38 | 0.3 |
| Nitrate | mg/ L | <0.1-0.5 | <0.1-0.5 | <0.1-0.5 | <0.001-2.15 | 50 |
| pH | - | 6.2– 7.3 | 6–7.5 | 6–7.17 | 5.5–6.92 | 6.5–8.5 |
| Potassium | mg/ L | 0.4– 5.9 | 1.8–3.7 | 1.2– 4.2 | 0.345–7.59 | n/a |
| Total alkalinity | mg/ L | 77.4– 324.2 | 154.4– 347.4 | 81.06– 393 | 125–341 | n/a |
| Total hardness | mg/ L | 149.5– 383 | 181.8– 686.6 | 113.1– 363.6 | 99.58– 419.08 | 500 |
| Sodium | mg/ L | 6.6– 84.7 | 4.3– 118.2 | 2.73– 39.6 | 0.425– 60.72 | n/a |
| Sulfate | mg/ L | 1–71 | 1–75 | 1–26 | 0–31 | 250 |

** NDWS is Nepal's drinking water standard. Agricultural standards, which are not yet available, may differ from NDWS. n/a is not available.*

In the Bhajani and Chuha sections of the Kailali district, Gurung et al. (2015) evaluated drinking water quality from groundwater. The metrics for colour, pH, turbidity, TDS, EC and total hardness are all within the National Standard for drinking water quality, according to an analysis of 24 groundwater samples. Some samples had elevated levels of heavy metals such as Al, Pb, Cd, Fe, As, and Mn, which may pose a concern to the local population. Although the region's water quality criteria for agricultural use have not yet been established, they are anticipated to differ from those for drinking.

**SM-4**: Estimated water use/demand and associated statistics in the river basins within the FtF-ZoI (Source: Pandey et al., 2010)

| River basin | Irrigation use (MCM) | Domestic use (MCM) | Total use (MCM) | Water use (% of WR) | Agricultural area (%) | Irrigation area (%) |
| --- | --- | --- | --- | --- | --- | --- |
| Mahakali | 450.96 | 26.49 | 477.45 | 2.64 | 23.26 | 29.86 |
| Karnali | 2,983.31 | 62.44 | 3,045.75 | 6.91 | 5.91 | 37.63 |
| Babai | 2,144.45 | 17.03 | 2,161.48 | 77.20 | 19.96 | 51.08 |
| West Rapti | 353.20 | 20.81 | 374.01 | 11.69 | 44.13 | 30.78 |

**SM-5(a)**: Agriculture and irrigation-related indicators in selected districts within the Sudurpashchim and Lumbini Provinces (Source: District profile published by Ministry of Agriculture and Livestock Development)

| Indicator | Dang district | Banke district | Kailali district |
| --- | --- | --- | --- |
| Proportion of population in agriculture-related occupation (%) | *A*griculture (20.7%); salaried/wage agriculture (3.0%) | *A*griculture (35.4%); salaried/wage agriculture (3.0%) | *A*griculture (23.8%); salaried/wage agriculture (3.4%) |
| Major sources of drinking water | Piped water (67.8%), open well (13.3%), and hand pump/tube well (8.3%) | Hand pump/ tube well (93.1%), piped water (6.6%) | Hand pump/ tube well (85.1%), piped water (12.4%) |
| Major sources of irrigation | Continuous flow canal (63.9% for irrigated agriculture land, 78.1% for temporary crops), tube well/boring (13.9% for irrigated agriculture land, and 5.3% for temporary crops) | Others (47.1% for irrigated agriculture land, 37.0% for temporary crops); continuous flow canal (25.1% for irrigated agriculture land, 20.7% for temporary crops), tube well/boring (17.9% for irrigated agriculture land, 16.3% for temporary crops) | Continuous flow canal (37.6% for irrigated agriculture land, 24.7% for temporary crops), tube well/boring (36.0% for irrigated agriculture land, and 50.6% for temporary crops) |
| Major cropping patterns in Khet land | Rice-Wheat-Fallow (31.6%); Rice-Wheat-Maize (23.0%); Rice-Maize-Fallow (13.0%); Rice-Rice-Wheat (12.2%) | Rice-Wheat-Fallow (48.9%); Rice-Fallow-Fallow (14.6%); Rice-Wheat-Maize (9.7%) | Rice-Wheat-Fallow (39.1%); Others (38.2%); Rice-Fallow-Fallow (7.5%) |
| Major cropping patterns in Bari land | Maize-Tori-Fallow (86.4%) | Others (63.5%); Maize-Rice-Wheat (9.0%); Maize-Tori-Fallow (7.5%) | Vegetable-Vegetable (49.2%); Others (31.8%); Vegetable-Maize (13.2%) |

**SM-5 (b)**: Supporting data for estimating irrigation water demand (Source: various, as indicated inside the Table itself)

| Source | Name of Report/ Information | Description of information | Limitation/Gap |
| --- | --- | --- | --- |
| Central Bureau of Statistics (CBS, 2011) | National Sample Census of Agriculture 2011/12. | In Province 5, Source of irrigation is 22% by gravity, 13% by pumping, 27% by dam/reservoir, 33% by tubewell/boring, 3% by others and 1% by mixed. In Province 7, source of irrigation is 52% by gravity, 5% by pumping, 12% by dam/reservoir, 28% tubewell/boring, 2% of others and 1% by mixed source of irrigation. | Didn’t find in the context of district but it has been stated by some report. |
| Ministry of Agricultural and Livestock Development. | District profile of Dang, Banke, Kailali district based on baseline survey of 2015. | District wise agriculture occupation, source of drinking water, irrigation water, cropping pattern of in Khet land and Bari land. | Only available for three Terai FtF district. |
|  | Frequently asked question on agriculture | Few information can be found related to crop irrigation system like for Rice, 2-3 cm of water should be maintained during sowing. After 3 days of sowing 5 cm of water should be maintained. During cultivation and fertilization, up to 5 days’ water should not applied. During budding and fruiting 10 cm of irrigation. Before 10-15 days of harvesting water should be not in farm. | As per the irrigation information of rice, the irrigation for other crop has not been explain in detail. This report is only available in Nepali version. |
| Ministry of Agriculture and Livestock Development (MoALD, 2020) | Statistical Information on Nepalese Agriculture(2018/19). | Agricultural production, Crop command area. | _ |
| IMP (2019) | Current and planned Cropping Intensity (CI) | For the baseline of 2019, CI: 132%, Target: 182% (upto 2025); 205% (2030); 230% (2045). | Only in context of Nepal, not district or province. |
|  | Karnali Transfer (New scheme transfer) | Target for 40,000 ha of irrigation by 2029-2035. |  |
|  | Naumure Dam, Kapilbastu transfer (new scheme transfer) | Target for 42,000 ha of irrigation by 2027 to 2033 |  |
|  | Bheri-Babai (completion of construction) | Current irrigation to 33,000 ha and target for 40,000 ha by 2025. |  |
|  | Rani Jamara (Completion of construction) | Current irrigation to 10,000 ha and target for 30,000 ha by 2025. |  |
|  | Mahakali III (Completion of construction) | Current irrigation to 5,000 ha and target for 25,000 ha by 2025. |  |
|  | Year Round Irrigation | 39% (2019), 60% (2025), 80% (2030), 100% (2045). |  |
|  | Irrigation Efficiency | More than 50% |  |
| ICIMOD (2010) GIS database | Agricultural command area | Six Terai districts within FtF-ZoI |  |
| IMP (2019) GIS database | Surface Irrigation NCA from shape file database | Six Terai districts within FtF-ZoI | No such irrigation command area by groundwater. |

**SM 5(c)**: Areas under agriculture and irrigation in Terai districts within Sudurpashchim and Lumbini Provinces (Source: total agricultural command areas from ICIMOD (2010) and other information from IMP (2019)

| District | Areas under agriculture (ha) | | | Areas under surface irrigation (NCA, ha) |
| --- | --- | --- | --- | --- |
|  | Total | Rice | Wheat |  |
| Kapilbastu | 96,255 | 58,000 | 31,000 | 15,915 |
| Dang | 119,497 | 36,508 | 12,655 | 27,548 |
| Banke | 65,191 | 31,900 | 18,022 | 25,076 |
| Bardia | 69,876 | 48,500 | 19,500 | 34,921 |
| Kailali | 102,457 | 71,250 | 34,500 | 29,554 |
| Kanchanpur | 61,981 | 45,796 | 31,433 | 16,874 |
| Total | 515,257 | 291,954 | 147,110 | 149,890 |

***SM-6****: Status of Surface- and Groundwater Access in FtF-ZoI*

**A) Status of surface water irrigation**:

There are 2.265 million ha of land in Nepal that can be used for irrigation, and 1.594 million ha (or 65 percent) of the total area) are in the Terai region. There are irrigation facilities on about 310,260 ha of land in the FtF-ZoI districts (Terai alone), which vary amongst the FtF-ZoI districts, as shown in Table 2. 231 surface irrigation schemes are operating in FtF-ZoI districts (Terai only), according to the inventory report created by the Irrigation Master Plan (IMP, 2019) (please refer to Annex 2-1 in Pandey et al. (2021), for details on those schemes). These schemes cover total gross and net irrigation command areas of 224,461 ha and 149,890 ha, respectively. All Farmer Managed Irrigation Systems (FMIS), Agency Managed Irrigation Systems (AMIS), and Agency-Farmer Managed Irrigation Systems are included in this statistic (AFMIS). **Figure S-6-1** displays the distribution of irrigation command areas and the locations of those irrigation schemes' headworks across the FtF-ZoI districts. Most irrigation plans have gross command areas between 12 and 6,000 ha, which are FMISs. In addition to FMISs, the federal and provincial governments are responsible for six significant irrigation projects in the FtF-ZoI areas (exclusively Terai). In addition, the FtF-ZoI districts (Terai alone) contain seven lift irrigation schemes, the management of which is entrusted to the provincial government. Of these, four (4) are located in Banke and three (3) in Kapilbastu districts (**Table 1, main manuscript**).

There is still no irrigation available for nearly 30% of the world's arable land. After realizing the value of year-round irrigation (YRI), the Government of Nepal (GoN) plans to undertake the multipurpose interbasin water transfer project (IBWTP) as per IMP (2019), which will move water from rivers with water surpluses to those with water shortages. Six (really six scenarios with four projects) of Nepal's eleven inter-basin water transfer projects are found in the FtF-ZoI districts. IBWTPs, also known as multifunctional projects, are frequently large-scale in terms of potential irrigated areas, infrastructure, and expenses. They also typically include hydropower generating. The donor catchment's water supply constraints the maximum irrigable area for IBWTPs.


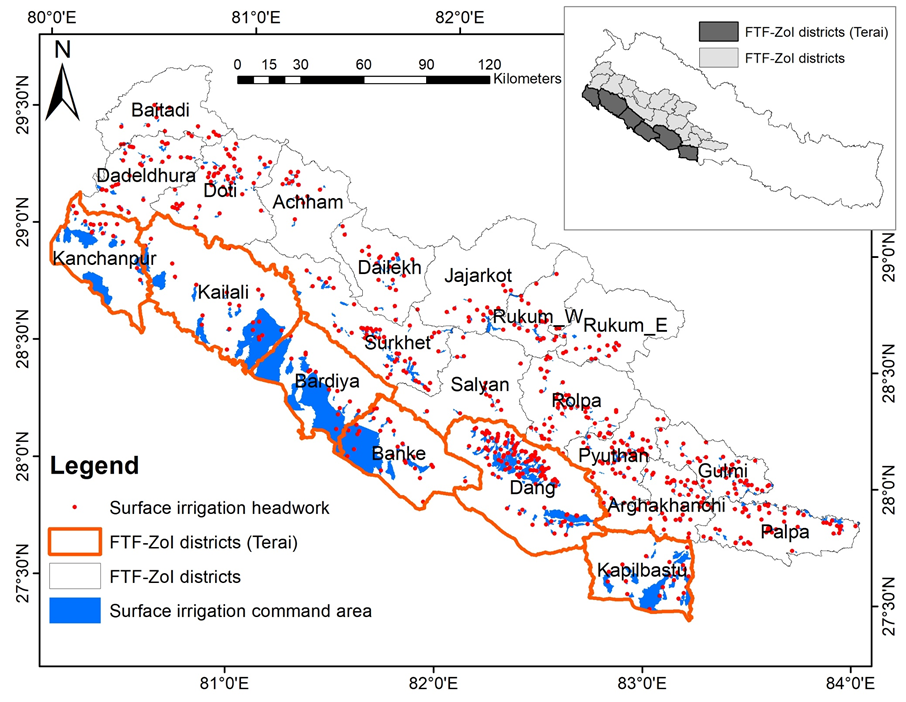


**Figure S-6-1**: Existing irrigation projects (surface) within the FtF-ZoI districts (Source: Pandey et al., 2021).

The rough geography and financial and technical limitations make it challenging to carry out river diversion or storage projects in the lower and middle hills. Hill irrigation raises additional issues such as insufficient irrigation coverage, a smaller YRI coverage area, inefficient water use, and underutilization of established infrastructure. In addition to the IBWTP, lift irrigation systems are also proposed for the FtF-ZoI to address those issues by pumping water from river courses that run parallel to agricultural areas. The Bheri Corridor Irrigation Development Project, Bheri Ghatgau - II Irrigation Project, Kusetara Irrigation Project, and Integrated Energy and Irrigation Special Program are notable lift irrigation systems proposed in the area.

Solar energy is being examined as a viable source of power for certain districts of the FtF-ZoI in Western Nepal's lift irrigation projects. Solar energy has the benefit of having low operating expenses because it typically operates when sunshine is present. The issue is that electricity output varies during the day based on solar intensity, which could change the water supply to fields used for irrigation. The following lists the solar-lift irrigation systems IMP (2019) proposed for the area.

**B) Status of groundwater irrigation**:

Based on the irrigation water use inventory report, the groundwater irrigation status in the FtF-ZoI districts (Terai only) is summarized (IMP, 2018). The report understates the true scope of groundwater irrigation in the Terai since it does not include privately installed tubewells that have grown rapidly and only documents government-funded tubewell projects. In the FtF-ZoI districts (Terai alone), there are approximately 40,939 STWs and 454 DTWs, which provide irrigation water to 73,253 ha and 12,550 ha, respectively, according to the inventory report (IMP, 2018). (**Table 1**). The Community Groundwater Irrigation Project, Community Shallow Tubewell Irrigation Project, Bhairahawa Lumbini Groundwater Irrigation Project, Nepal Irrigation Sector Project, etc., are only a few the important groundwater irrigation projects the government has started. Most STWs are built to irrigate 1 hectare of land at a time. About 28 percent of all irrigated land is supplied by groundwater irrigation systems, with differences between FtF-ZoI districts (Terai alone) ranging from 13 percent (in Bardia) to 53 percent (in Kanchanpur). Kanchanpur (53 percent), Kailali (41 percent), Banke (19 percent), Dang (17 percent), Kapilbastu (17 percent), and Bardiya (13 percent) are the districts in decreasing order of gross command area (GCA) irrigated with groundwater. Nearly 85 percent of the groundwater-irrigated regions get their water from STW, and the remaining 15 percent from DTW. The percentage of STW used for total groundwater irrigation varies by district, ranging from 93 percent in Kailali to 61 percent in Dang (**Table 1**).

Regarding upcoming groundwater irrigation projects, no precise information is available. The Sambriddha Terai Madhesh Irrigation Special Program is a significant government program that is now being implemented in this area. Based on the water shortage caused by surface irrigation projects across the districts, IMP (2019) depicts the planned groundwater irrigation regions. The concept is that groundwater will fill the irrigation shortfall areas where surface water is insufficient. There is no water shortage for the Bardia and Kanchapur districts, hence there are no plans for groundwater irrigation in the future for those two districts. However, it's unclear whether all available surface water can be diverted for irrigating farmlands, even if it can, how long it will take and whether they can meet irrigation demands in time for agricultural needs, even if they can. Investments for groundwater irrigation must therefore be focused in such areas as well.

To address the surface irrigation system's irrigation shortage, planned groundwater development must be prioritised in the Kapilbastu and Dang districts. There are only a few hundred hectares of groundwater irrigation in the districts of Kapilbastu and Dang. By the end of the planning period, more areas may require groundwater supply either because more areas will be coming under irrigation, the efficiency of surface water systems will be decreasing, or other water use sectors' demands will be rising.

**References**

Bhatta, B., Shrestha, S., Shrestha, P. K. & Talchabhadel, R., (2020). Modelling the impact of past and future climate scenarios on streamflow in a highly mountainous watershed: A case study in the West Seti River Basin, Nepal. *Science of the Total Environment, Journal Pre-proof.* <https://doi.org/10.1016/j.scitotenv.2020.140156>.

Bohara, M. S. (2015). Physico-Chemical and Microbiological Analysis of Drinking Water Quality of Bhim Datta Muncipality of Kanchanpur. *Journal of Microbiological Research,* 1(1): 1-7.

CBS, (2011). Province Summary of Agriculture Census 2011. Available at: <https://cbs.gov.np/province-summary-of-agriculture-census-2011/>. [Assessed 21 9 2020]

Dahal, P., Shrestha, M. L., Panthi, J. & Pradhananga, D. (2020). Modeling the future impacts of climate change on water availability in the Karnali River Basin of Nepal. Environmental Research, 185: 109430. <https://doi.org/10.1016/j.envres.2020.109430>.

Dhami, B., Himanshu. S. K., Pandey., A. & Gautam, A. K. (2018). Evaluation of the SWAT model for water balance study of a mountainous snowfed river basin of Nepal. *Environmental Earth Sciences, 77(21): 1-20.* <https://doi.org/10.1007/s12665-017-7210-8>.

GDC, 1994. Reassessment of the Groundwater development strategy for irrigation in the Terai (Volume 3 – Groundwater). Groundwater Development Consultants Ltd. (GDC). April, 1994. Submitted to the Government of Nepal, Department of Irrigation, Groundwater Resources Development Project.

Gurung, S., Raut, N., Shrestha, S., Gurung, J., Maharjan, B. & Shrestha, S. (2015). Assessment of Groundwater Quality in Far Western Kailali District, Nepal. *Jacobs Journal of Hydrology,* 1(1): 1-9.

IMP (2018). Irrigation water use inventory – final report. Irrigation Master Plan (IPM), Preparation through Integrated River Basin Planning. Prepared by Lahmeyer International in May 2018 for Water Resources Project Preparation Facility, Department of Irrigation, Ministry of Irrigation, Government of Nepal

IMP, (2019). Irrigation Master Plan Preparation through Integrated River Basin Planning, 2019; Prepared for Water Resources Project Preparatory Facility Department of Irrigation Ministry of Irrigation Nepal; Prepared by Tractebel Engineering GmbH in association with NIRAS and TMS.

Khatiwada, K. R., Panthi , J., Shrestha, M. L. & Nepal, S. (2016). Hydro-Climatic Variability in the Karnali River Basin of Nepal Himalaya. *MDPI,* 74(6): 1-14.

Mahat, K. R. & Shrestha, R. (2008). Metal Contamination in Ground Water of Dang District. *Nepal Journal of Science and Technology,* 9: 143-148

Mishra, Y., Nakamura, Tai., Babel, M.S., Ninsawat, S. & Ochi, S. (2019). Impact of Climate Change on Water Resources of the Bheri River Basin, Nepal. MDPI, 220(10): 1-21.

MoALD, (2015a). *District Profile Banke,* Singha Durbar, Kathmandu: Government of Nepal, Ministry of Agricultural Development, URL: <http://www.namis.gov.np/>.

MoALD, (2015b). *District Profile Dang,* Singha Durbar, Kathmandu: Government of Nepal, Ministry of Agricultural Development, URL: <http://www.namis.gov.np/>.

MoALD, (2015c). *District Profile Kailali,* Singha Durbar, Kathmandu: Government of Nepal, Ministry of Agricultural Development, URL: <http://www.namis.gov.np/>.

MoALD/GoN (2020). Statistical information on Nepalese agriculture 2075/76 [2018/19]. Statistics and Analysis Sector, Planning and Development Cooperation Coordination Division, Ministry of Agriculture & Livestock Development (MoALD), Government of Nepal (GoN). Singha Durbar, Kathmandu, Nepal.

MoALD/GoN (2020). Statistical information on Nepalese agriculture 2075/76 [2018/19]. Statistics and Analysis Sector, Planning and Development Cooperation Coordination Division, Ministry of Agriculture & Livestock Development (MoALD), Government of Nepal (GoN). Singha Durbar, Kathmandu, Nepal.

Pandey, V. P., Babel, M. S., Shrestha, S. & Kazama, F. (2010). Vulnerability of freshwater resources in large and medium Nepalese river basins to environmental change. *Water Science & Technology*, 61(6): 1525-1534.

Pandey, V. P., Dhaubanjar, S., Bharati, L. & Thapa, B. R. (2019). Hydrological response of Chamelia watershed in Mahakali Basin, 650 (1): 365-383. <https://doi.org/10.1016/j.scitotenv.2018.09.053>

Pandey, V. P., Dhaubanjar, S., Bharati, L. & Thapa, B. R. (2020, b). Spatio-temporal distribution of water availability in KarnaliMohana Basin, Western Nepal: Climate change impact assessment (Part-B). *Journal of Hydrology: Regional Studies.* <https://doi.org/10.1016/j.ejrh.2020.100691>.

Pandey, V. P., Ray, A., Khadka, M., Urfels, A., McDonald, A., & Krupnik, T. J. (2021). *Towards Conjunctive Use of Surface Water and Groundwater Resources as a Response to Water Access Challenges in the Western Plains of Nepal*. Retrieved from Kathmandu, Nepal: <https://csisa.org/wp-content/uploads/sites/2/2021/06/Water-Access-Report_Nepal-June2021-Web-rev.pdf>

Pathak, D., 2018. Status of Groundwater Exploitation And Investigation In Terai And Inner Terai. *Bulletin of Nepal Hydrogeological Association,*3: 77-83.

Shrestha SR, Tripathi GN and Laudari D (2018), Groundwater Resources of Nepal: An Overview. In Groundwater of South Asia (pp 169-193), Springer, Singapore.

Shrestha, N. (2017). *Projection of Futures Streams Flow And Their Uncertainty West Rapti Basin, Nepal*. Master Thesis University of Twente, Netherlands.

Tripathee, N. (2018). Climate Change Impacts on Water Resources and Crop Yield in Mohana River Basin, Nepal. Master of Engineering in Water Engineering Management (Unpublished Thesis). Asian Institute of Technology, School of Engineering and Technology.

WECS, (2011). Water Resources of Nepal in the Context of Climate Change. Singha Durbar, Kathmandu, Nepal: Water and Energy Commission Secretariat.

WHO, (2020). Arsenic. [Online] Available at: <https://www.who.int/news-room/fact-sheets/detail/arsenic>. [Accessed 22 October 2020].

Yadav, I. C., Singh, S., Devi, N.L., Mohan, P. M., Tater, P. S. & Shakya, B. M. (2012). Spatial Distribution of Arsenic in Groundwater of Southern Nepal. *Reviews of Environmental Contamination and Toxicology*,125-139.
